# FetalSegNet: Automatic Segmentation of the Fetus and Sub-Anatomical Structures in Prenatal Ultrasound Images Using Hybrid Deep Learning Models

**DOI:** 10.3390/diagnostics16213607

**Published:** 2026-11-01

**Authors:** Hilal Gülsüm Turan Özsoy, Eda Nur Urtekin, Furkan Ertürk Urfalı, Gültekin Adanaş Aydın, Ayşe Füsun Bekirçavuşoğlu, Emre Dandıl

**Affiliations:** 1 Department of Radiology, Bursa City Hospital, 16250 Bursa, Türkiye; drfurkanurfali@gmail.com (F.E.U.); aysefusunt@gmail.com (A.F.B.); 2 Department of Computer Engineering, Faculty of Engineering, Bilecik Şeyh Edebali University, 11100 Bilecik, Türkiye; urtekinedanur77@gmail.com; 3 Department of Obstetrics and Gynecology, Bursa City Hospital, 16250 Bursa, Türkiye; gadanas@gmail.com

**Keywords:** prenatal ultrasound, multi-label segmentation, fetal segmentation, deep learning, EfficientNetV2, UNet++, explainable artificial intelligence

## Abstract

**Background/Objectives**: Automatic segmentation of fetal anatomical structures in prenatal ultrasound images is challenging because of low tissue contrast, noise caused by speckles, anatomical variability and the fact that structures are only partially visible within individual image frames. This study has developed and evaluated FetalSegNet, a multi-label deep learning framework that can simultaneously perform segmentation of the fetus and selected sub-anatomical structures, including the head, arms, legs and heart. **Methods**: A dataset comprising 3321 prenatal ultrasound images obtained from 217 patients was created, with pixel-level annotations generated under expert supervision. FetalSegNet was implemented using four hybrid encoder–decoder configurations that combine EfficientNetV2-M and MaxViT-S encoders with UNet++ and Feature Pyramid Network (FPN) decoders. Performance was evaluated on an unseen test set using Dice score, intersection over union (IoU), precision, recall, 95th percentile Hausdorff distance (HD_95_) and average symmetric surface distance (ASSD). The presence of anatomical structures was also assessed using classification metrics, while HiResCAM was used to examine the spatial relevance of model activations. **Results**: Among the FetalSegNet configurations, the EfficientNetV2-M-UNet++ model achieved the highest overall segmentation performance, with a Dice (DSC) score of 0.9232, IoU of 0.8746, HD_95_ of 4.23 mm, and ASSD of 1.02 mm. Class-specific results indicated better performance in segmentation for the head and fetus, whereas the leg and heart classes showed comparatively poorer and more inconsistent results. The models also achieved high F1-scores and a high level of accuracy in assessing the presence of anatomical structures. HiResCAM analyses showed that model activations were predominantly concentrated within the corresponding anatomical regions. **Conclusions**: FetalSegNet provides a unified, multi-label framework for simultaneously segmenting the fetus and multiple sub-anatomical structures in prenatal ultrasound images. By combining a hybrid deep-learning architecture with independent output-mask channels, comprehensive boundary-based evaluation, and activation-based interpretability analysis, the framework offers an integrated strategy for fetal anatomical segmentation. These findings support further investigation of FetalSegNet for automated and interpretable analysis of prenatal ultrasound images.

## 1. Introduction

Prenatal ultrasound (US) imaging is an essential tool in routine prenatal care. It is widely used to assess fetal growth, estimate gestational age, evaluate fetal anatomy and detect potential abnormalities [1]. US is particularly suitable for repeated examinations during pregnancy due to its real-time imaging capability, relative cost-effectiveness, portability, and absence of ionizing radiation [2]. When the necessary expertise and equipment are available, second-trimester prenatal ultrasound examinations can improve gestational age assessment and the detection of fetal anomalies, while enabling the assessment of major fetal anatomical regions, including the head, face, spine, chest, heart, abdomen and extremities [3].

Despite its widespread use, the interpretation of prenatal US remains strongly influenced by the quality of image acquisition and the operator’s experience [4]. This issue is particularly important in routine clinical practice, where the position of the fetus, gestational age, maternal and fetal factors, acoustic shadowing, movement and variations in acquisition protocols can all have a significant impact on image quality and diagnostic visualization [5,6,7]. These difficulties emphasize the importance of scalable, automated image analysis technologies that can reliably identify and evaluate fetal anatomical structures in different imaging conditions, especially in environments where specialist expertise and advanced imaging resources are scarce.

From an image analysis perspective, prenatal ultrasound raises particularly challenging segmentation issues. Unlike many anatomical structures visualized with other medical imaging modalities, fetal structures may have poorly defined boundaries and heterogeneous echogenicity. Challenges include speckle noise, low tissue contrast, acoustic shadows, reverberation, motion artefacts and variations in fetal position or orientation, all of which can make the boundary between the target anatomy and surrounding tissues difficult to identify [8]. Furthermore, fetal anatomical structures may be incompletely visualized and may exhibit considerable variation in apparent morphology across imaging planes and fetal positions. Consequently, conventional image-processing and rule-based segmentation approaches often lack the flexibility and robustness required to generalize across patients, imaging devices, operators, and acquisition conditions [9,10,11]. A comprehensive review of deep learning applications in fetal ultrasound has highlighted variations in image appearance, anatomical morphology and acquisition conditions, as well as the limited availability of annotated data, as important challenges for automated fetal image analysis [12]. These challenges are particularly important for multi-structure segmentation, since the anatomical regions visible in each image can vary in size, appearance and spatial distribution.

The development of deep learning has transformed the automated analysis of fetal ultrasound images. U-Net-based encoder–decoder architectures have been widely adopted for biomedical image segmentation due to their strong performance in learning hierarchical image representations directly from data [13,14]. Subsequent adaptations of U-Net have been extensively applied to fetal ultrasound segmentation, including the delineation of the fetal head, abdomen, and other anatomical structures. U-Net-based models and their variants have reported high overlap scores for fetal head and abdominal segmentation, supporting the feasibility of deep learning for automated fetal biometry and anatomical delineation [15,16]. Previous research on medical image segmentation has investigated integrating multi-stage preprocessing, U-Net-based architectures and attention mechanisms to support the delineation of anatomical structures [17].

The emergence of more complex architectures, such as nested and multi-scale designs, has further enhanced the ability of segmentation models to identify fine anatomical structures. For example, UNet++ introduces nested skip pathways that reduce the semantic gap between the feature maps of the encoder and decoder, and provide more flexible feature aggregation [18,19]. Similarly, feature pyramid networks (FPNs) exploit hierarchical feature representations at multiple spatial scales, making them well-suited to structures that exhibit substantial variation in size and appearance [20,21]. These architectures are particularly relevant in the context of fetal ultrasound, where regions such as the head, limbs, heart and whole fetus differ considerably in terms of spatial scale and visual characteristics [22,23].

Recently, transformer-based and hybrid CNN-transformer architectures have introduced more effective mechanisms for modelling long-range spatial dependencies. In addition to the design of the decoder, the architectures of the encoders differ in their approaches to feature extraction and modelling of spatial dependency. For example, MaxViT combines local block attention with global dilated grid attention within a hierarchical architecture, enabling interactions between fine-grained and global image features while maintaining linear computational complexity with respect to image size [24]. Another relevant development is EfficientNetV2, which combines efficient convolutional operations with training-aware neural architecture search to produce strong feature representations while improving parameter and computational efficiency [25]. Together, these advances suggest that encoder selection plays an important role in extracting complementary local and global information from challenging ultrasound images.

Despite the rapid development of deep-learning methods for fetal ultrasound analysis, the existing literature remains substantially fragmented. A systematic review found that research has focused predominantly on the fetal head, followed by the body, heart, face, and abdomen, with classification and segmentation representing the most frequently investigated computer-vision tasks [12,26]. Fetal-ultrasound applications have broadly been grouped into three categories: standard-plane detection, anatomical-structure analysis, and biometric estimation [27]. However, persistent challenges—including limited and heterogeneous datasets, labor-intensive annotation, uncertain cross-device and cross-center generalizability, and the translation of research models into clinical practice—continue to constrain clinical adoption [28,29].

Although many previous studies have reported promising results for individual target structures, optimizing a model for a single structure does not necessarily satisfy the broader requirements of comprehensive fetal-image assessment [30]. During clinical ultrasound examinations, multiple anatomical regions may be visible simultaneously, and their spatial relationships can provide valuable contextual information. For example, a model trained exclusively to segment the fetal head cannot directly characterize the fetal body, limbs, or heart within the same image. Training separate models for different structures also increases computational and deployment complexity and may produce fragmented analysis pipelines. This limitation is particularly important when structures overlap, exhibit substantial scale variation, or undergo marked appearance changes across ultrasound examinations. Another important point to consider is that anatomical structures of the fetus are not necessarily mutually exclusive in an ultrasound image. Depending on the imaging plane and the extent of visible anatomy, the fetal region may be annotated alongside individual structures, such as the head, arms, legs or heart. According to their annotation boundaries, these independently defined regions may share overlapping pixels. Therefore, traditional multiclass semantic segmentation, where each pixel is assigned to a single class, may not accurately reflect the underlying anatomical organization. When annotations overlap, a mutually exclusive multiclass approach requires each pixel to be assigned to only one category. This can result in information associated with other annotated structures being discarded. A multi-label framework with separate binary mask channels offers a more flexible representation, as each anatomical structure can be modelled individually while permitting spatial overlap between target regions. In this approach, each channel represents the presence or absence of its corresponding anatomical structure at pixel level independently. Consequently, a pixel can belong to multiple annotated regions without the labels needing to be merged or assigned a fixed priority. This enables the segmentation targets to retain their original annotation structure while avoiding mutual exclusivity between anatomical categories.

The availability and diversity of annotated datasets remain a major challenge. While several publicly available fetal ultrasound datasets have facilitated progress in specific tasks, many studies still rely on relatively small datasets, single anatomical targets or datasets acquired under narrowly defined imaging protocols. Consequently, there is still a need for datasets that include multiple fetal anatomical structures, as well as models that can exploit such annotations within a unified segmentation framework. In addition to segmentation accuracy, explainability is important when segmenting fetal anatomical structures. High numerical performance alone does not demonstrate that a model has learned anatomically meaningful features, particularly in ultrasound, where predictions may be influenced by acquisition-related or background characteristics rather than clinically relevant anatomy. Explainable artificial intelligence (XAI) methods can provide complementary insight by highlighting image regions that contribute to model predictions. HiResCAM was introduced as a class-specific explanation method that produces spatially precise attribution maps and addresses a limitation of Grad-CAM caused by spatial gradient averaging [31].

With this research background, this study proposes a unified, multi-label, deep-learning framework for the simultaneous segmentation of the fetus and multiple sub-anatomical structures in prenatal ultrasound images. The study focuses specifically on the fetal region, head, arms, legs and heart. A dataset is constructed and annotated at pixel level under expert supervision. Rather than forcing the anatomical categories into mutually exclusive classes, each target structure is represented by an associated binary mask. Accordingly, the segmentation models generate five output channels, with each one providing an independent estimate of the probability of a pixel belonging to a specific anatomical structure. This approach is designed to allow for spatial overlap and enable the simultaneous prediction of multiple anatomical regions. To examine the impact of various feature extraction and reconstruction strategies, two compatible encoder architectures (EfficientNetV2-M and MaxViT-S) are combined with two decoder designs (UNet++ and FPN), resulting in four hybrid segmentation models. This experimental design allows for a controlled comparison of convolutional and attention-enhanced feature representations, as well as intertwined and multi-scale decoder structures. In addition, the presence of anatomical structures is evaluated separately, and HiResCAM is used to examine whether the learned representations are concentrated spatially on the corresponding anatomical regions. The main contributions of this study can therefore be summarized as follows:

- We propose a unified, multi-label fetal ultrasound segmentation framework that can simultaneously delineate the fetus, head, arms, legs and heart. This represents a broader approach to anatomical segmentation than the single-structure focus commonly encountered in previous studies.
- To accommodate spatial overlap between fetal anatomical structures and prevent the need for each pixel to belong to only one anatomical category, a multi-label annotation strategy based on independent binary mask channels is employed.
- A comparative analysis of convolutional, attention-based, stacked and multi-scale feature representations for fetal ultrasound segmentation is provided by systematically investigating four hybrid encoder–decoder architectures, which combine EfficientNetV2-M and MaxViT-S encoders with UNet++ and FPN decoders.
- A comprehensive segmentation evaluation is performed using both overlap- and boundary-based metrics, which enables a more detailed characterization of the model’s behavior.
- Anatomical structure presence/absence analysis and a HiResCAM-based assessment of explainability are used to determine whether the models produce correct segmentations and whether their learned responses are spatially associated with the expected anatomical regions.

## 2. Materials and Methods

**Figure 1:**
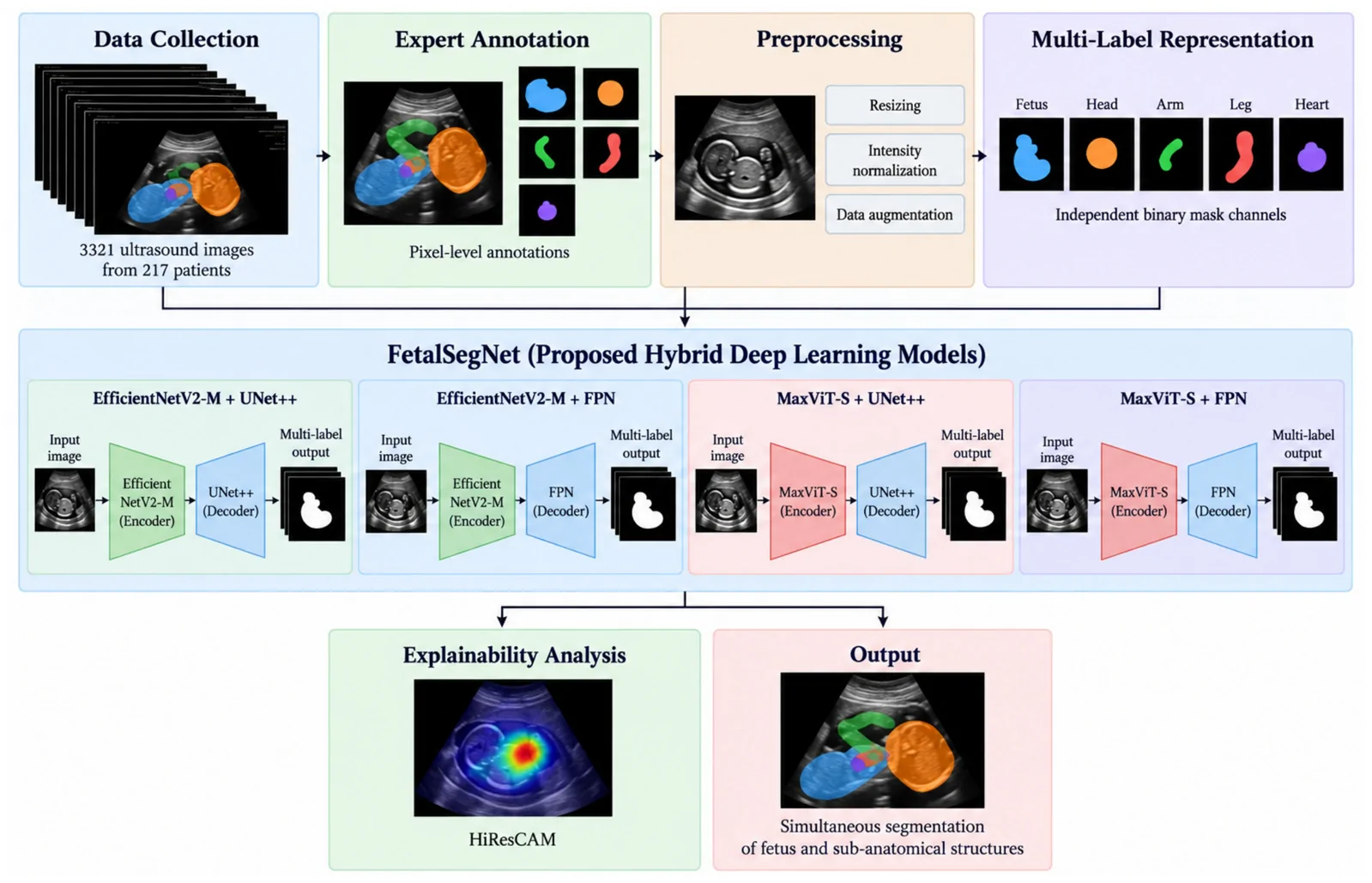
Overall workflow of the proposed FetalSegNet framework for multi-label segmentation of fetal and sub-anatomical structures in prenatal ultrasound images.

Figure 1 shows the overall workflow of the proposed FetalSegNet framework. The framework used prenatal ultrasound images obtained from 217 patients. Pixel-level annotations were generated for the fetus, head, arms, legs and heart under expert supervision. The images were subjected to predefined pre-processing procedures, including resizing, intensity normalization and data augmentation. In order to account for the individual representation of anatomically separate and potentially overlapping structures, each target was represented by a distinct binary mask channel. FetalSegNet was developed as a hybrid encoder–decoder framework by combining EfficientNetV2-M and MaxViT-S as alternative encoders, and UNet++ and FPN as alternative decoders. This resulted in the construction of four configurations: EfficientNetV2-M + UNet++, EfficientNetV2-M + FPN, MaxViT-S + UNet++ and MaxViT-S + FPN. The encoder extracts hierarchical feature representations from the input ultrasound images, while the decoder reconstructs spatial information to generate multi-label segmentation maps. Each configuration was trained separately using annotated data and then evaluated using key performance metrics. The presence of anatomical structures was additionally assessed using the F1-score and accuracy. Finally, HiResCAM was used to investigate the spatial regions contributing to the model’s predictions and to provide an interpretable evaluation of the learned representations. The resulting framework generates segmentation maps simultaneously for both the fetus and the targeted sub-anatomical structures.

### 2.1. Dataset

The dataset was retrospectively constructed from routine prenatal ultrasound examinations performed at Bursa City Hospital, Bursa, Türkiye. It included 217 singleton pregnancies involving women aged 18–40 years, with a mean maternal age of 25.94 ± 4.83 years and a median age of 26 years. The gestational age at examination ranged from 15 to 24 weeks, and examinations were conducted between July 2025 and July 2026. Only pregnancies with normal fetal development were included; cases with known major fetal abnormalities were excluded. All examinations were performed in the Department of Radiology at Bursa City Hospital using a Voluson E8 ultrasound system (GE Healthcare, Zipf, Austria) equipped with a C2–9 MHz convex transducer [32]. Rather than using individual still images acquired directly from the scanner, prenatal ultrasound videos recorded during routine examinations served as the source data, from which individual frames were extracted.

The number of extracted frames varied substantially between pregnancies, ranging from approximately 91 to 1457 frames. No fixed temporal sampling interval was used; instead, an experienced radiologist visually reviewed the extracted frames and selected diagnostically appropriate fetal views. To limit redundancy, between 1 and 106 unique frames were retained per pregnancy, with a median of 10 frames per patient. Pixel-level MD5 hashing was subsequently used to identify and remove duplicate images, ensuring that frames with identical pixel content were not treated as independent observations. All selected frames were independently reviewed by an experienced fetal-imaging radiologist in collaboration with an obstetrician specializing in prenatal ultrasound. For each eligible image, the fetus, head, arm, leg, and heart were manually delineated at the pixel level to generate reference segmentation masks. The annotation process was supervised by the obstetrician to ensure anatomical consistency and accurate delineation of the target structures, resulting in a final dataset of 3321 image-mask pairs.

Table 1 summarizes the dataset according to patient, image, and anatomical class. Before processing, all images were de-identified by removing potentially identifiable patient information. The study complied with the ethical principles of the Declaration of Helsinki and applicable personal-data-protection regulations. Ethical approval was obtained from the Scientific Research Ethics Committee of Bursa City Hospital (Decision No. 2024-21/22; 11 December 2024), and written informed consent was obtained from all participating pregnant women before inclusion.

**Figure 2:**
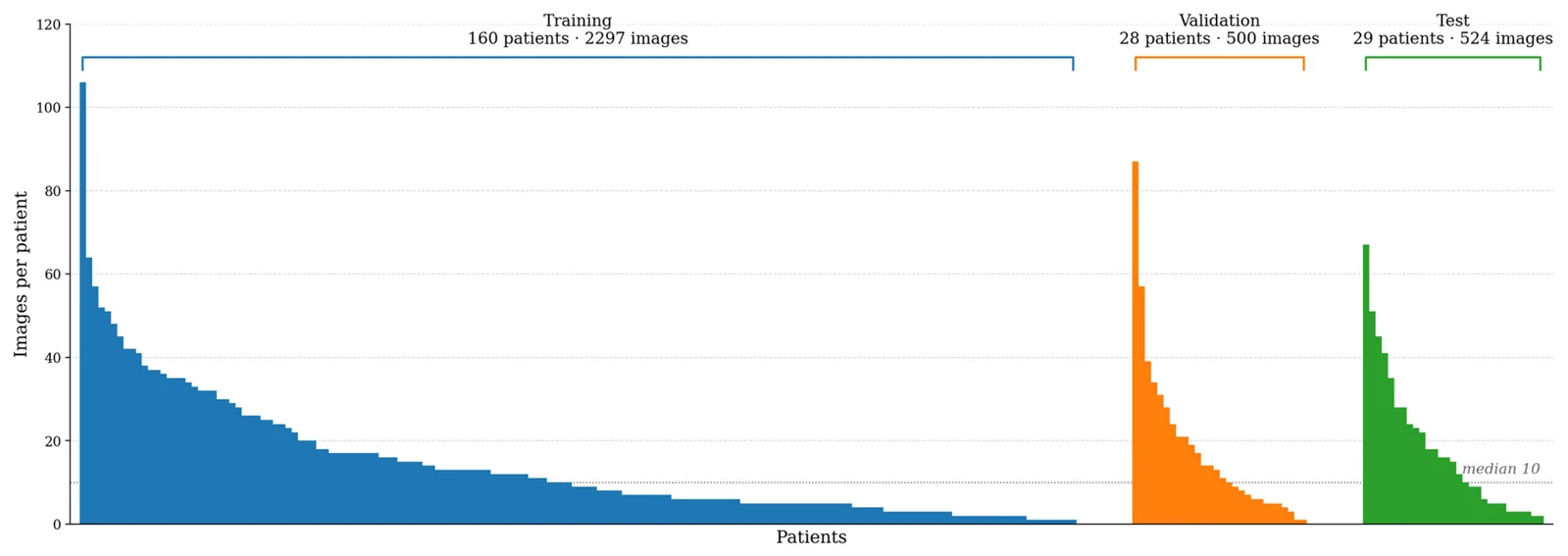
The patient-level dataset partition of images per patient across the training, validation and test subsets.

As can be seen in Figure 2, the dataset was divided at the patient level, ensuring that all of a patient’s frames were assigned to a single subset and preventing any patient from appearing in more than one subset. To maintain a comparable anatomical class distribution across the subsets, a class-balanced procedure was used to allocate patients: each patient was placed into the subset whose current composition was furthest below the target 70:15:15 ratio when considering the total number of images and the number of images containing each anatomical structure (fetus, head, arm, leg and heart). The resulting training, validation and test subsets are mutually exclusive at the patient level and approximately preserve the per-class frequencies.

**Figure 3:**
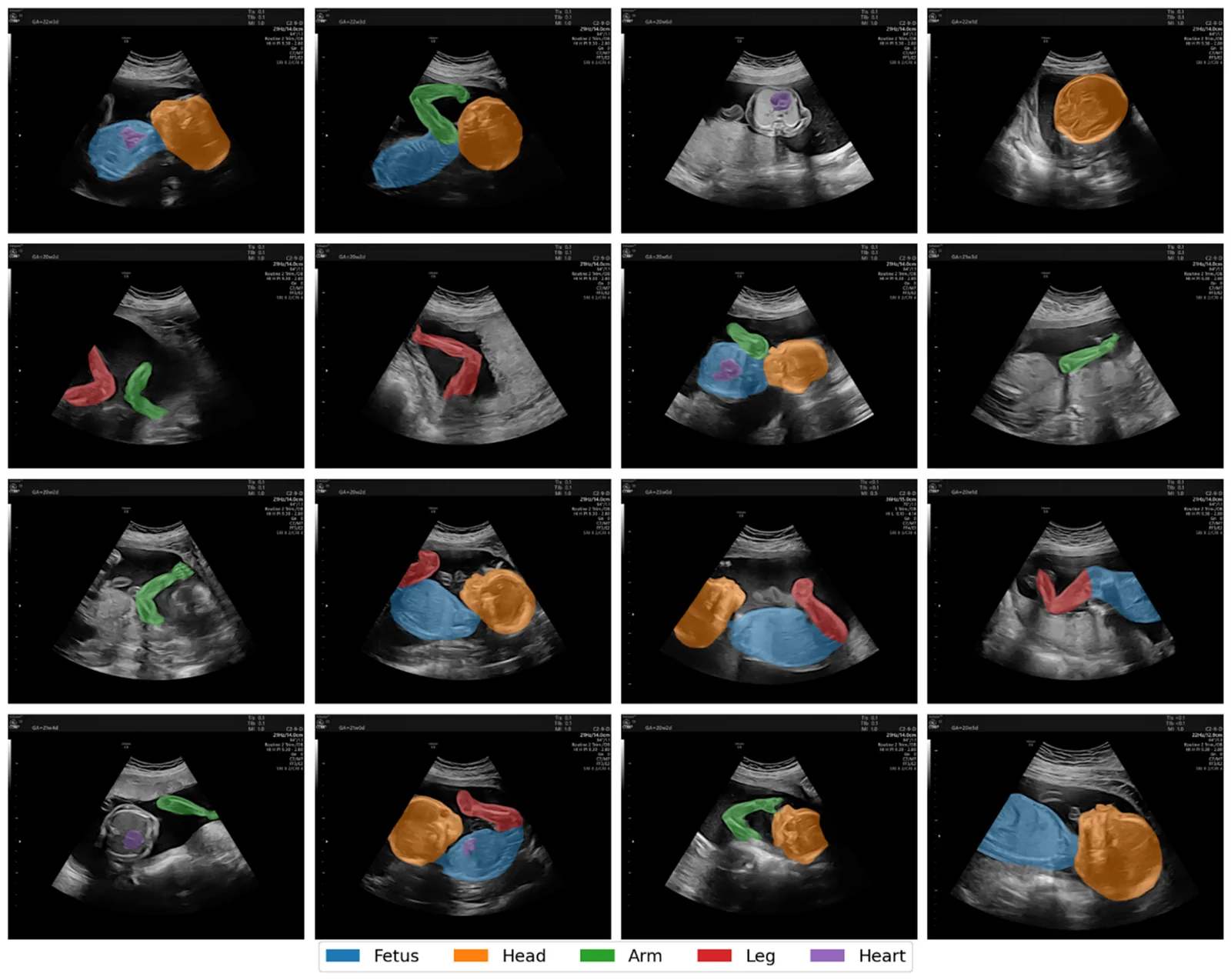
Representative samples from the prenatal ultrasound dataset showing pixel-level annotations of the fetus and sub-anatomical structures.

Figure 3 shows examples of images from the prenatal ultrasound dataset, together with their corresponding pixel-level annotations. These examples demonstrate significant variability in fetal orientation, anatomical appearance, imaging plane and visibility of individual structures across examinations. According to the annotation scheme, the fetus, head, arm, leg and heart are shown in blue, orange, green, red and purple, respectively. The number and visibility of annotated structures vary between images according to fetal position and imaging plane. Multiple structures may be visible simultaneously, lying in close proximity to one another or overlapping, which highlights the challenges of multi-structure segmentation in prenatal ultrasound. The examples also demonstrate variations in image quality and anatomical scale, as well as the extent to which structures are captured within the field of view. Consequently, these characteristics collectively represent the heterogeneity addressed by the proposed FetalSegNet framework.

### 2.2. Pre-Processing and Data Augmentation

Before the models were trained and evaluated, the prenatal ultrasound images were processed using a standardized pipeline consisting of intensity normalization and architecture-compatible input preparation. Data augmentation was applied exclusively during training. These processes were implemented to enhance the consistency of the input data and facilitate the learning of consistent anatomical representations from ultrasound images.

In order to meet the input configuration requirements of the proposed FetalSegNet framework, the original ultrasound images (with a spatial resolution of 864 × 1136 pixels) were processed using zero-padding rather than resizing, in order to preserve their anatomical geometry and boundary information. The padding dimensions were determined according to the spatial constraints of the encoder architectures. EfficientNetV2-M requires input dimensions divisible by 32 due to its hierarchical down-sampling operations. In contrast, the MaxViT-S configuration requires dimensions divisible by 224 to accommodate its 7 × 7 window and grid attention operations at a feature stride of 32. Accordingly, the images were padded to 864 × 1152 pixels for EfficientNetV2-M and to 896 × 1344 pixels for MaxViT-S without altering the original content of the images. After spatial preparation, the grayscale image intensities were normalized to the [0, 1] range and replicated across three channels to match the input requirements of the pre-trained encoders. Intensity normalization was applied to maintain a consistent numerical input range across ultrasound images acquired under different imaging conditions. The corresponding segmentation masks underwent the same spatial padding as their respective images to preserve pixel-level alignment. These preprocessing operations were consistently applied to the training, validation and test sets, while data augmentation was restricted to the training set.

The augmentation pipeline combined geometric and appearance-related transformations to address various sources of variability in prenatal ultrasound images, including fetal orientation, anatomical positioning, intensity differences and variations in image quality. Instead of using fixed transformation combinations, each transformation was sampled independently according to its specified probability, enabling multiple transformations to be applied sequentially to the same training sample. The dataset was not physically expanded by storing copies of the augmented images. Instead, data augmentation was performed dynamically and randomly during training, enabling the models to encounter different augmented versions of the same training samples across epochs. To ensure that model selection and final performance evaluation were conducted on data that had not been subjected to training-time augmentation, no data augmentation was applied to the validation or test sets. Data augmentation was only applied during training to improve the models’ generalization capability and expose them to variations in prenatal ultrasound images. This was implemented using the Albumentations library [33]. Simultaneously, geometric transformations were applied to both the ultrasound images and their corresponding anatomical mask channels in order to preserve pixel-level alignment. The geometric augmentation pipeline included horizontal flipping with a probability of 0.5, vertical flipping with a probability of 0.3, and a combined shift-scale-rotate transformation with a probability of 0.4. For the latter transformation, translation was limited to ±5%, scaling to ±10%, and rotation to ±10°. Elastic deformation was also applied as a geometric transformation with α = 60 and σ = 6 (*p* = 0.25). These transformations were selected using moderate, commonly adopted augmentation settings rather than a separate parameter optimization procedure. This was done with the aim of introducing spatial variability while limiting excessive alterations to anatomical geometry and boundaries. Photometric and image-quality transformations included brightness and contrast modification (±0.15; *p* = 0.40), Gaussian blurring using kernel sizes of 3–5 (*p* = 0.20) and Gaussian noise with a variance range of 10–50 (*p* = 0.20). Brightness and contrast adjustments were included to vary image intensity, whereas blurring and the addition of noise were used to introduce occasional image-quality degradation. These transformations were assigned moderate ranges and application probabilities to increase image diversity without obscuring anatomical details consistently. Unlike geometric transformations, intensity-based and noise transformations were applied only to the ultrasound images, leaving their corresponding segmentation masks unmodified.

**Figure 4:**
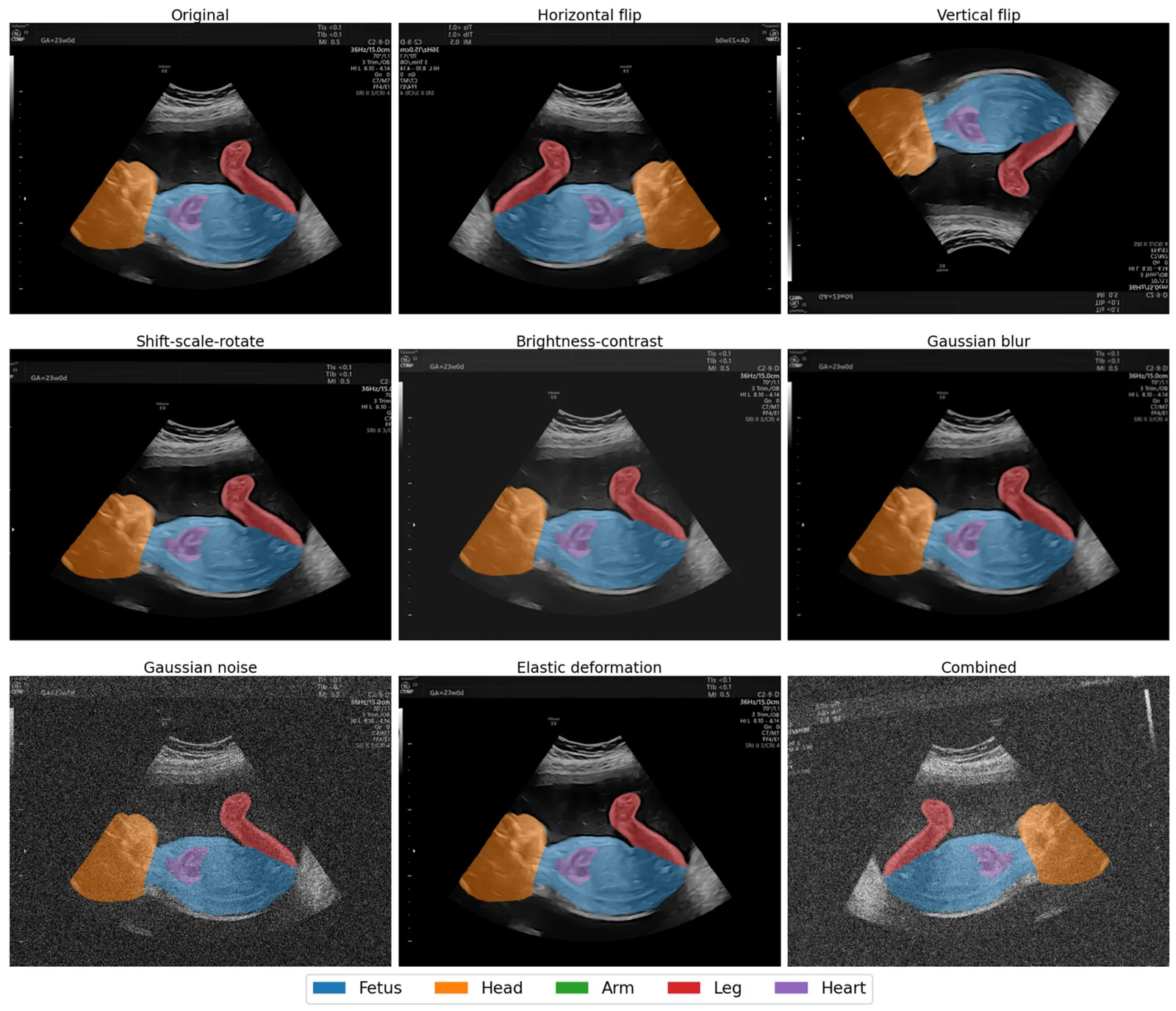
Some examples of geometric and photometric data augmentation applied to prenatal ultrasound images during model training.

Figure 4 shows some of the data augmentation transformations that were applied during model training. These include horizontal and vertical flipping, shift–scale–rotate transformations, brightness–contrast adjustment, Gaussian blurring and noise addition, and elastic deformation. The final combined example shows the outcome of applying multiple transformations sequentially. The anatomical annotations are preserved and transformed in conjunction with the corresponding ultrasound images.

### 2.3. Proposed Hybrid Deep Learning Architectures

FetalSegNet (Fetal Segmentation Network) is a hybrid encoder–decoder framework designed for simultaneous multi-label segmentation of the fetus and its sub-anatomical structures in prenatal ultrasound images. The framework is based on the principle that effective medical-image segmentation requires both discriminative feature extraction and accurate spatial reconstruction. Accordingly, these functions are separated into an encoder and a decoder, allowing different feature-extraction and reconstruction strategies to be integrated systematically.

**Figure 5:**
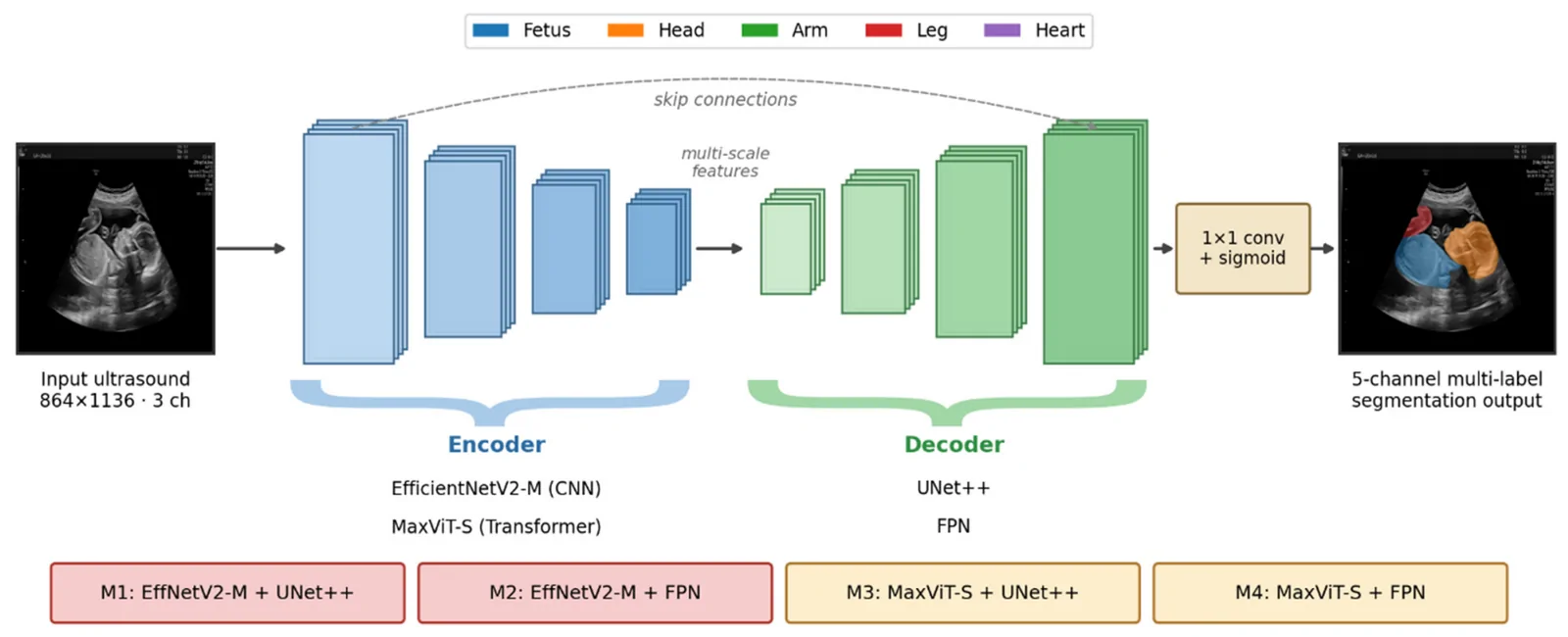
Overall architecture and workflow of the proposed FetalSegNet framework.

In FetalSegNet, the encoder extracts hierarchical and multi-scale representations from the input ultrasound image. The decoder then progressively transforms these representations into pixel-level segmentation maps. To investigate the contribution of convolution-based and hybrid convolution-attention representations, two complementary encoder architectures were selected: EfficientNetV2-M and MaxViT-S. At the decoder level, UNet++ and the Feature Pyramid Network (FPN) were chosen as they offer distinct approaches to recovering spatial information and integrating features across resolutions. Combining these four encoder–decoder combinations resulted in four hybrid segmentation configurations: EfficientNetV2-M + UNet++, EfficientNetV2-M + FPN, MaxViT-S + UNet++ and MaxViT-S + FPN. The complete workflow is illustrated in Figure 5.

The central rationale of FetalSegNet is to combine complementary architectural properties rather than rely on a single encoder or decoder design. Prenatal ultrasound images contain fine-grained local information, such as anatomical boundaries and tissue interfaces, as well as broader contextual information related to fetal position and inter-structural spatial relationships. EfficientNetV2-M provides hierarchical convolutional feature extraction with an emphasis on computational efficiency, whereas MaxViT-S complements these representations with local and global attention mechanisms. At the decoding stage, UNet++ and FPN provide distinct strategies for spatial reconstruction. UNet++ uses nested, densely connected skip pathways to progressively reduce the semantic gap between encoder and decoder representations, while FPN combines features across resolutions through a top-down pathway and lateral connections. Their combination with the two encoders produces four configurations: EfficientNetV2-M + UNet++, EfficientNetV2-M + FPN, MaxViT-S + UNet++, and MaxViT-S + FPN. This design is well suited to multi-label fetal segmentation, in which anatomical structures vary substantially in size, appearance, visibility, and spatial context. The four configurations were therefore evaluated under identical experimental conditions to compare their segmentation performance and interpretability.

#### 2.3.1. Encoder Architectures

The EfficientNet family was developed in response to the observation that scaling the depth, width and input resolution of a network in a balanced manner can lead to a more effective allocation of computational resources [34]. EfficientNetV2 is a family of convolutional neural networks designed to balance predictive accuracy, parameter efficiency, and training speed. The architecture was developed using training-aware neural architecture search and scaling, with an expanded search space that includes operations such as Fused-MBConv [25]. Compared with the original EfficientNet family, EfficientNetV2 introduces several architectural modifications—including the selective use of Fused-MBConv blocks, smaller expansion ratios, and revised stage configurations—to improve training efficiency while preserving strong representational capacity. In the proposed FetalSegNet framework, EfficientNetV2-M acts as the feature-extraction encoder. Its hierarchical representations extract information at various semantic levels, ranging from local edges and textures in the initial stages to more abstract anatomical features in the subsequent stages. This multi-level representation is particularly well-suited to fetal ultrasound segmentation, where target structures can vary considerably in size and appearance. For example, the fetus and head may occupy relatively large regions, whereas the arms, legs and heart may appear smaller or only be partially visible. Rather than being used as a complete image-classification network, EfficientNetV2-M is used as a backbone. Therefore, its classification head is removed and its intermediate feature maps are passed to the corresponding decoder stages. This enables the backbone to operate as a hierarchical encoder within the encoder–decoder architecture proposed, thereby preserving spatially informative features for pixel-level segmentation.

The second encoder used in FetalSegNet is the MaxViT-S. This was developed from the MaxViT to address the challenge of modelling both local and long-range spatial relationships efficiently in high-resolution images [24]. Its architecture combines convolutional operations with a multi-axis attention mechanism consisting of blocked local attention and dilated global attention. This design enables the network to model both local and global interactions, while also maintaining a more scalable computational structure than if global self-attention were applied directly over all image locations. This study is particularly relevant to prenatal ultrasound, where the interpretation of a local anatomical region may depend on its spatial relationship with the surrounding fetal structures. For instance, a limb or cardiac region may appear ambiguous in isolation, but become clearer when contextual information from neighbouring structures is considered. Accordingly, MaxViT-S complements the predominantly convolutional EfficientNetV2-M encoder by providing explicit local-global context modelling through multi-axis attention. Similarly, in FetalSegNet, the MaxViT-S classification head is removed and its hierarchical feature maps are passed to the selected segmentation decoder. Therefore, MaxViT-S serves as the attention-enhanced encoder component of the proposed hybrid framework.

#### 2.3.2. Decoder Architectures

The first decoder architecture used in FetalSegNet is UNet++, which was proposed for medical image segmentation [18]. UNet++ builds upon the traditional U-Net encoder–decoder model by introducing nested and dense skip pathways between the encoder and decoder subnetworks [19]. The main reason for this is to reduce the semantic gap between the features of the encoder and the features of the decoder before they are fused. In conventional U-Net, skip connections directly link feature maps from corresponding encoder and decoder levels [35]. Although these connections preserve high-resolution spatial information, the semantic representations at the two ends may differ substantially. UNet++ addresses this semantic gap by introducing intermediate convolutional nodes and nested, densely connected skip pathways, allowing encoder features to be progressively transformed and enriched before fusion with decoder representations [18]. This characteristic is advantageous for fetal ultrasound segmentation because precise boundary delineation requires the integration of high-level semantic information with fine-grained spatial detail. UNet++ facilitates this integration by progressively reducing the semantic discrepancy between encoder and decoder representations through its nested skip pathways. Its original architecture also incorporates deep supervision, allowing intermediate decoder outputs to contribute to model training. In FetalSegNet, UNet++ is used as the decoder with both EfficientNetV2-M and MaxViT-S, producing two configurations: EfficientNetV2-M + UNet++ and MaxViT-S + UNet++. This design enables the contribution of the nested decoder to be evaluated alongside both a convolution-based encoder and an attention-enhanced encoder.

The second decoder is based on the Feature Pyramid Network (FPN), which was originally introduced as an architecture for representing features in object detection. It was designed to exploit the multi-scale hierarchy inherent in deep convolutional networks [20]. It generates semantically enriched representations at multiple spatial resolutions by constructing a feature pyramid through a top-down pathway and lateral connections. The main advantage of FPN is its ability to combine high-level semantic information with lower-level spatial detail at various scales [36]. Although it was originally developed for object detection, FPN’s multi-scale feature fusion principle is also well-suited to dense prediction tasks, such as semantic segmentation [37]. In the present study, FPN is employed as a lightweight, multi-scale decoder that transforms hierarchical encoder representations into segmentation features. This design is particularly relevant to the current task, as the target structures exhibit significant differences in spatial scale and visibility. For example, the fetus and head may occupy relatively large regions, whereas the heart and limbs can occupy substantially smaller regions that are only partially visible. FPN is separately combined with both encoder alternatives to produce the EfficientNetV2-M + FPN and MaxViT-S + FPN configurations. Consequently, using FPN provides an alternative decoder strategy to the nested skip pathways of UNet++.

## 3. Results

Comprehensive analysis of the experimental results of the four FetalSegNet configurations (EfficientNetV2-M + UNet++, EfficientNetV2-M + FPN, MaxViT-S + UNet++ and MaxViT-S + FPN) is provided. First, the results describe the training and validation behavior of the models, followed by their quantitative segmentation performance on the independent test set. Both overall and class-specific results are reported for the fetus, head, arm, leg and heart classes, using both region-overlap and pixel-level metrics. Boundary accuracy is also assessed using surface-distance metrics. As the multi-label dataset contains images with various combinations of visible anatomical structures, the results also include an analysis of the presence of anatomical structures based on specificity and F1-score. Representative qualitative segmentation examples are then presented to illustrate the predicted masks across different ultrasound appearances. Finally, explainability results based on HiResCAM are reported to visualize the spatial regions associated with the model predictions. The findings are presented systematically, highlighting the observed quantitative and qualitative performance of the FetalSegNet configurations.

To ensure a consistent computational environment across all experiments, all model training, tests and explainability analyses were performed on a single GPU. The models were implemented in Python 3.9.25 using PyTorch 2.5.1 and TorchVision 0.20.1. The segmentation architectures were developed using the segmentation_models_pytorch (0.5.0) and timm (1.0.25) libraries, and Albumentations (2.0.8) was employed for image pre-processing and data augmentation. NumPy (2.0.2) was used for numerical array operations, image and segmentation-mask manipulation, pixel-level calculations, and the aggregation of quantitative evaluation metrics. All experiments were conducted on an NVIDIA GeForce RTX 4070 Ti SUPER GPU with 16 GB of memory. This was supported by an AMD Ryzen 5 7600 CPU and 64 GB of system RAM. This system ran on Ubuntu 24.04.4 LTS with CUDA 12.1 and cuDNN 9.1. The GPU driver provided CUDA 12.8 compatibility.

The original grayscale ultrasound images were converted into three-channel representations and normalized to the [0, 1] range for model input. As the two encoder architectures required different spatial input dimensions, zero-padding was only applied to the right and bottom edges of the original images, which were 864 × 1136 pixels in size. This resulted in input dimensions of 864 × 1152 pixels for EfficientNetV2-M and 896 × 1344 pixels for MaxViT-S. The padding was introduced solely to satisfy the spatial input requirements of the encoder architectures and did not represent additional image content. Consequently, during training and evaluation, loss calculations, segmentation metrics and visualization procedures were performed with reference to the original 864 × 1136 pixel field of view, excluding the artificially padded regions.

To ensure consistent and fair comparisons between the FetalSegNet configurations, the same training protocol and optimization parameters shown in Table 2 were used for all models. The training criterion was defined as a weighted combination of E-Focal Loss and Dice Loss, with respective weights of 0.60 and 0.40. The model generated five independent output channels, each of which corresponded to a binary anatomical mask. These were then converted into pixel-level probabilities using a sigmoid function. These predictions were optimized against their respective reference masks using the combined loss function. E-Focal Loss evaluated prediction errors at a pixel level across the five anatomical channels, with class-specific focusing parameters that were dynamically adjusted according to the accumulated positive and negative gradient magnitudes [38]. Dice loss was computed in multi-label mode to measure the overlap between each predicted anatomical region and its respective reference mask [39]. Both loss components contributed to a single training objective, enabling the five channels to influence the optimization of the shared encoder–decoder parameters and segmentation head collectively. No explicit inter-channel anatomical consistency constraint or additional containment penalty was imposed on the predicted structures. The AdamW optimizer was used with an initial learning rate of 2 × 10^−4^, which was then adjusted using a cosine annealing strategy [40]. Each model was trained for a maximum of 150 epochs, with an early stopping patience of 25 epochs to prevent unnecessary training once validation performance ceased to improve. Due to GPU memory limitations associated with the relatively large input resolutions, a batch size of 2 was used physically. To achieve an effective batch size of 8, gradients were accumulated over four iterations prior to each optimizer update. These training settings were kept identical across all hybrid configurations to ensure that differences in model performance could be examined under a consistent optimization protocol. Mixed-precision training was implemented to enhance computational efficiency and mitigate GPU memory usage. Gradient accumulation was employed during training, and gradient clipping with a maximum gradient norm of 1.0 was applied to enhance stability and prevent excessive gradient updates. For the MaxViT-S-based configurations, gradient checkpointing was also activated to decrease memory usage during the forward and backward passes. The optimal model weights for each configuration were selected based on the highest mean Dice score achieved on the validation set. The independent test set was kept completely separate from the process of selecting the models and was only used for the final evaluation of the trained models. The encoders were initially set up using ImageNet pre-trained weights. A fixed random seed (42) was used for data splitting, weight initialization and augmentation to ensure reproducibility.

The performance of the proposed FetalSegNet segmentation framework was evaluated using a combination of region overlap, pixel-level classification and boundary distance metrics. This combination was chosen in order to assess both the agreement between the predicted and reference regions and the pixel-level errors, as well as the spatial accuracy of the anatomical boundaries. The Dice similarity coefficient (DSC) was used as the main metric for quantifying spatial overlap between the predicted (PM) and ground-truth/reference (GM) segmentation masks, as defined in Equation (1). The intersection over union (IoU), shown in Equation (2), is another overlap metric that was calculated to quantify the relative overlap between the PM and GM regions. As with DSC, higher IoU values signify better segmentation alignment.

(1) $$\mathrm{DSC}=\frac{2\left|\mathrm{PM}\cap \mathrm{GM}\right|}{\left|\mathrm{PM}\right|+\left|\mathrm{GM}\right|}$$

(2) $$\mathrm{IoU}=\frac{\left|\mathrm{PM}\cap \mathrm{GM}\right|}{\left|\mathrm{PM}\cup \mathrm{GM}\right|}$$

The performance of pixel-level classification was further assessed using Precision and Recall. Precision is the measure of the proportion of pixels predicted as positive that actually belong to the corresponding anatomical structure. Recall, on the other hand, is the measure of the proportion of reference-positive pixels that are correctly identified by the model. These metrics were obtained using Equations (3) and (4).

(3) $$\mathrm{Precision}=\frac{\mathrm{TP}}{\mathrm{TP}+\mathrm{FP}}$$

(4) $$\mathrm{Recall}=\frac{\mathrm{TP}}{\mathrm{TP}+\mathrm{FN}}$$

where TP, FP, and FN denote the numbers of True Positive, False Positive, and False Negative pixels, respectively.

As overlap-based measures may not fully characterize the accuracy of anatomical boundaries, boundary alignment was also evaluated using the 95th-percentile Hausdorff Distance (HD_95_) and the Average Symmetric Surface Distance (ASSD). These distance-based metrics provide additional information about the spatial displacement of the predicted anatomical boundaries in relation to the reference segmentation [41]. HD_95_ in Equation (7) was used to evaluate the geometric precision of the predicted segmentation boundaries. Equations (5) and (6) define the bidirectional point-to-surface Euclidean distances between PM and GM. The minimum distance to the opposing boundary is then calculated for each boundary point. HD_95_ is then obtained by taking the maximum of the two 95th-percentile distances in each direction. Unlike the conventional Hausdorff distance, which depends on the maximum boundary distance, HD_95_ minimises the impact of isolated outliers while maintaining sensitivity to significant boundary differences. ASSD metric in Equation (8) was used to quantify the average geometric difference between PM and GM segmentations. Unlike HD_95_, which describes the upper tail of the bidirectional surface distance distribution, the ASSD measures the mean symmetric distance between the corresponding segmentation boundaries. The minimum Euclidean distance from each point on PM boundary to GM boundary is calculated, and the same procedure is applied in the opposite direction. The two surface distances in each direction are then averaged to obtain the ASSD.

(5) $$d(x,\mathrm{GM})=\underset{y\in \mathrm{GM}}{\min}{\left\|x-y\right\|}_{2}$$

(6) $$d(y,\mathrm{PM})=\underset{x\in \mathrm{PM}}{\min}{\left\|y-x\right\|}_{2}$$

(7) $${\mathrm{HD}}_{95}\left(\mathrm{PM},\mathrm{GM}\right)=\max\left(P_{95}(d(\mathrm{PM},\mathrm{GM})),P_{95}(d(\mathrm{GM},\mathrm{PM}\right))$$

(8) $$\mathrm{ASSD}\;\left(\mathrm{PM},\mathrm{GM}\right)=\frac{\sum_{x\in \mathrm{PM}}d\;(x,\mathrm{GM})+\sum_{y\in \mathrm{GM}}d\;(y,\mathrm{PM})+}{\left|\mathrm{PM}\right|+\left|\mathrm{GM}\right|}$$

As the proposed FetalSegNet involves multi-label segmentation, not all target anatomical structures are present in every ultrasound image. Therefore, segmentation metrics were calculated for a given anatomical class only for matched images in which the corresponding structure was identified in both the reference annotation and the model prediction. This prevents regions with absent anatomical structures from dominating the region-overlap statistics artificially. Examples where a structure present in the anatomy was completely missed by the model, as well as examples where a structure absent from the reference annotation was incorrectly generated by the model, were evaluated separately as anatomical structure presence/absence classification. To this purpose, the numbers of True Negatives (TN), TP, FP and FN were used to calculate Specificity and the F1-score in Equation (9) and Equation (10), respectively.

(9) $$\mathrm{Specificity}=\frac{\mathrm{TN}}{\mathrm{TN}+\mathrm{FP}}$$

(10) $$F1-\mathrm{score}=\frac{2\times (\mathrm{Precision}\times \mathrm{Recall})}{\mathrm{Precision}+\mathrm{Recall}}$$

In order to report boundary-distance measurements in clinically interpretable physical units, the pixel spacing of each ultrasound image was estimated using the 1 cm depth-scale markings located along the left-hand side of the image. The number of pixels corresponding to each 1 cm interval was then used to calculate the pixel-to-millimeter conversion factor for each image individually. *HD*_95_ and ASSD were then calculated in pixel coordinates and converted to millimeters (mm) using the corresponding, image-specific pixel spacing. This procedure accounts for variations in the displayed depth scale between images and avoids the assumption of a single, global pixel-to-millimeter conversion factor.

**Figure 6:**
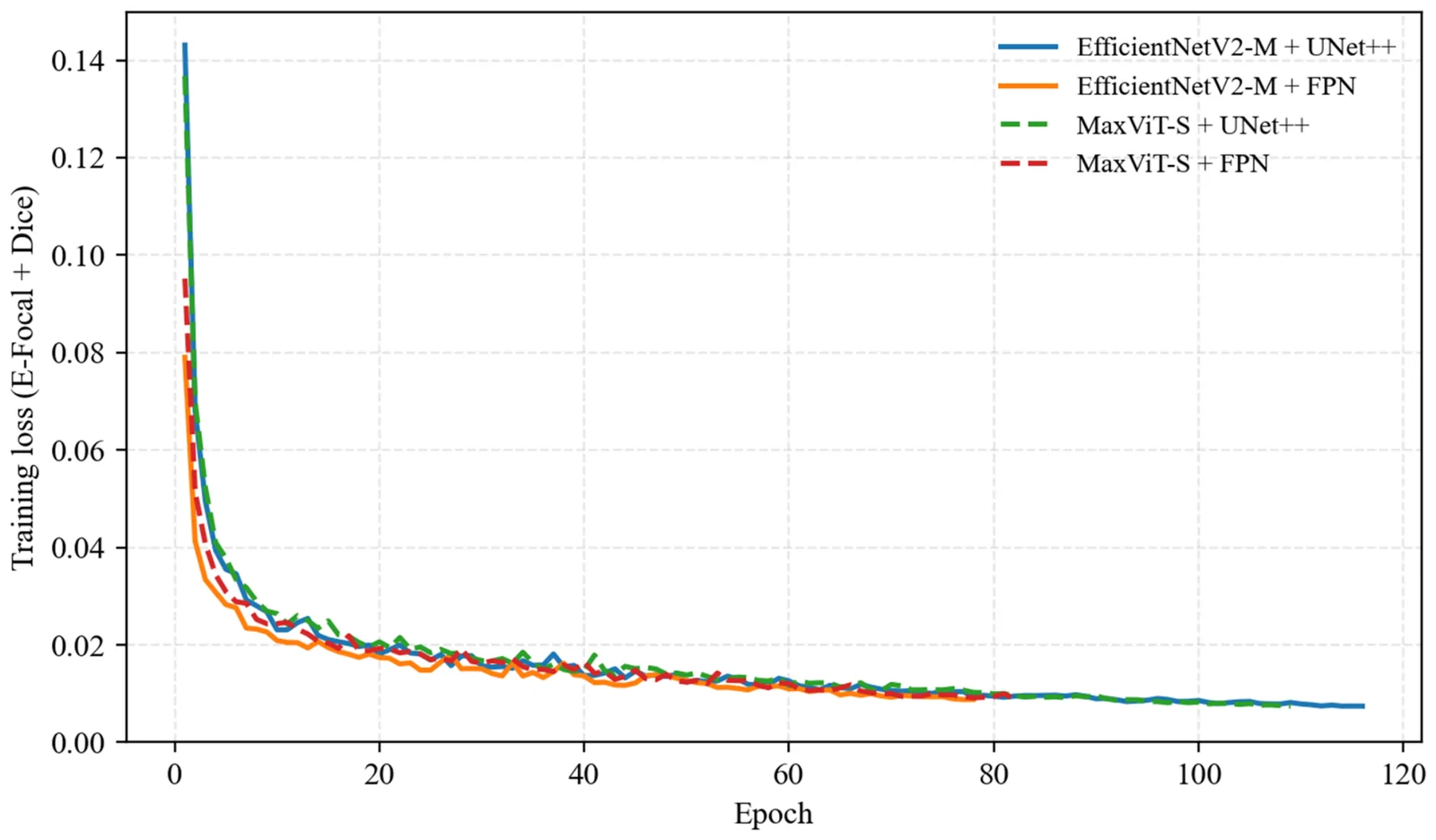
Training loss curves of the four FetalSegNet configurations across training epochs.

**Figure 7:**
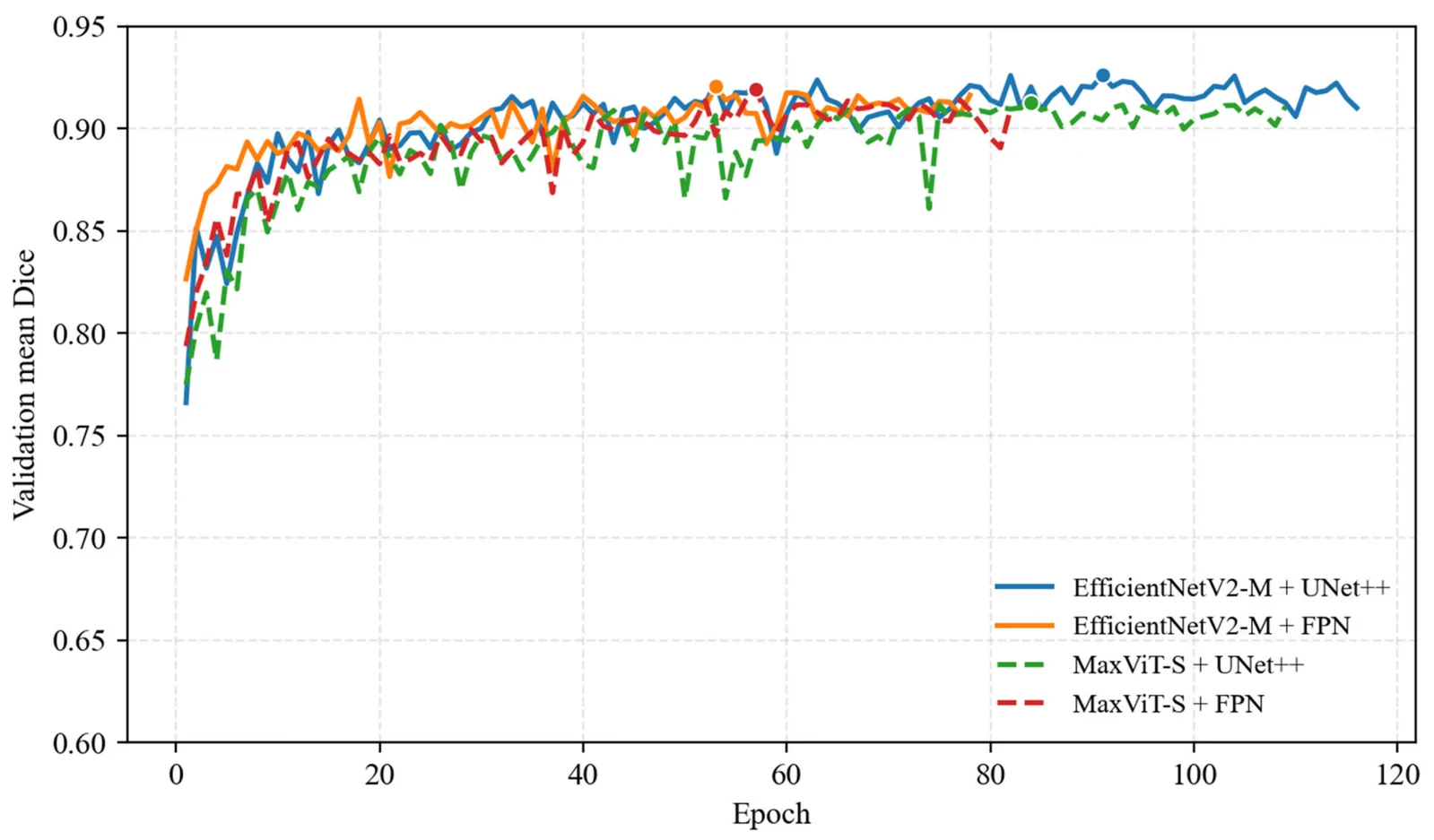
Mean validation Dice curves of the four FetalSegNet configurations across training epochs.

The learning behavior of the FetalSegNet configurations was investigated by observing the training loss and mean Dice score on the validation set during training. The relevant training and validation curves are presented in Figure 6 and Figure 7, respectively. As can be seen in Figure 6, all configurations exhibited a rapid decrease in training loss during the initial epochs, indicating the efficient acquisition of the main anatomical and spatial characteristics from the training data. Following this initial reduction, the loss curves gradually stabilized and continued to decrease at a substantially slower rate. Towards the end of the training process, the training losses of all configurations reached relatively low values, indicating that the models had reached a stable optimization stage. The validation Dice curves shown in Figure 7 revealed a consistent pattern. All configurations achieved a substantial improvement in Dice validation during the initial training phase, with the mean Dice score increasing. Subsequently, validation performance improved more gradually, fluctuating around the 0.90–0.92 range during the later epochs. Such fluctuations are to be expected in multi-label medical image segmentation, especially when anatomical structures vary considerably in size, appearance, visibility and image quality. The configurations behaved similarly in terms of convergence, although differences in the magnitude and frequency of validation fluctuations were observed. EfficientNetV2-M + UNet++ maintained consistently high validation Dice scores during the latter stages of training and achieved values above 0.90 for most of this period. The EfficientNetV2-M + FPN configuration exhibited a comparable convergence pattern, featuring gradual improvement followed by stable performance. The two MaxViT-S-based configurations also achieved high validation Dice scores, although larger fluctuations were observed between epochs, particularly during the intermediate training stages.

A summary of the training process is presented in Table 3. Of the four configurations, EfficientNetV2-M + UNet++ achieved the highest validation DSC score (0.9261) at epoch 91, indicating the best performance of the evaluated models during training. However, the EfficientNetV2-M + FPN and MaxViT-S + FPN configurations achieved their best validation performance earlier, at epochs 53 and 57 respectively, with DSC scores of 0.9210 and 0.9191. The MaxViT-S + UNet++ configuration achieved a validation DSC score of 0.9128 at epoch 84. The corresponding training losses at the selected epochs ranged from 0.0090 to 0.0117, with the lowest value (0.0090) achieved by EfficientNetV2-M + UNet++.

The final model weights were evaluated using an unseen test set of 524 images, which were not used during training or selection of the model. Table 4 reports the overall segmentation performance, averaged across all anatomical classes, using DSC, IoU, Precision, Recall, HD_95_ and ASSD. All four configurations achieved mean DSC and IoU scores above 0.89 and 0.83, respectively, on the test set, indicating consistent segmentation performance across the evaluated hybrid architectures. The EfficientNetV2-M + UNet++ configuration produced the best overall results, with DSC, IoU, Precision and Recall scores of 0.9232, 0.8746, 0.9393 and 0.9248 respectively. This configuration also produced the lowest boundary-distance errors, with an HD_95_ of 4.23 mm and an ASSD of 1.02 mm. The results show that the combination of region-overlap and boundary-based segmentation performance achieved by EfficientNetV2-M + UNet++ was the highest on the test set. Its higher DSC and IoU values were associated with the lowest HD_95_ and ASSD values, indicating that its improved regional overlap was also related to closer alignment between the predicted and reference anatomical boundaries.

**Figure 8:**
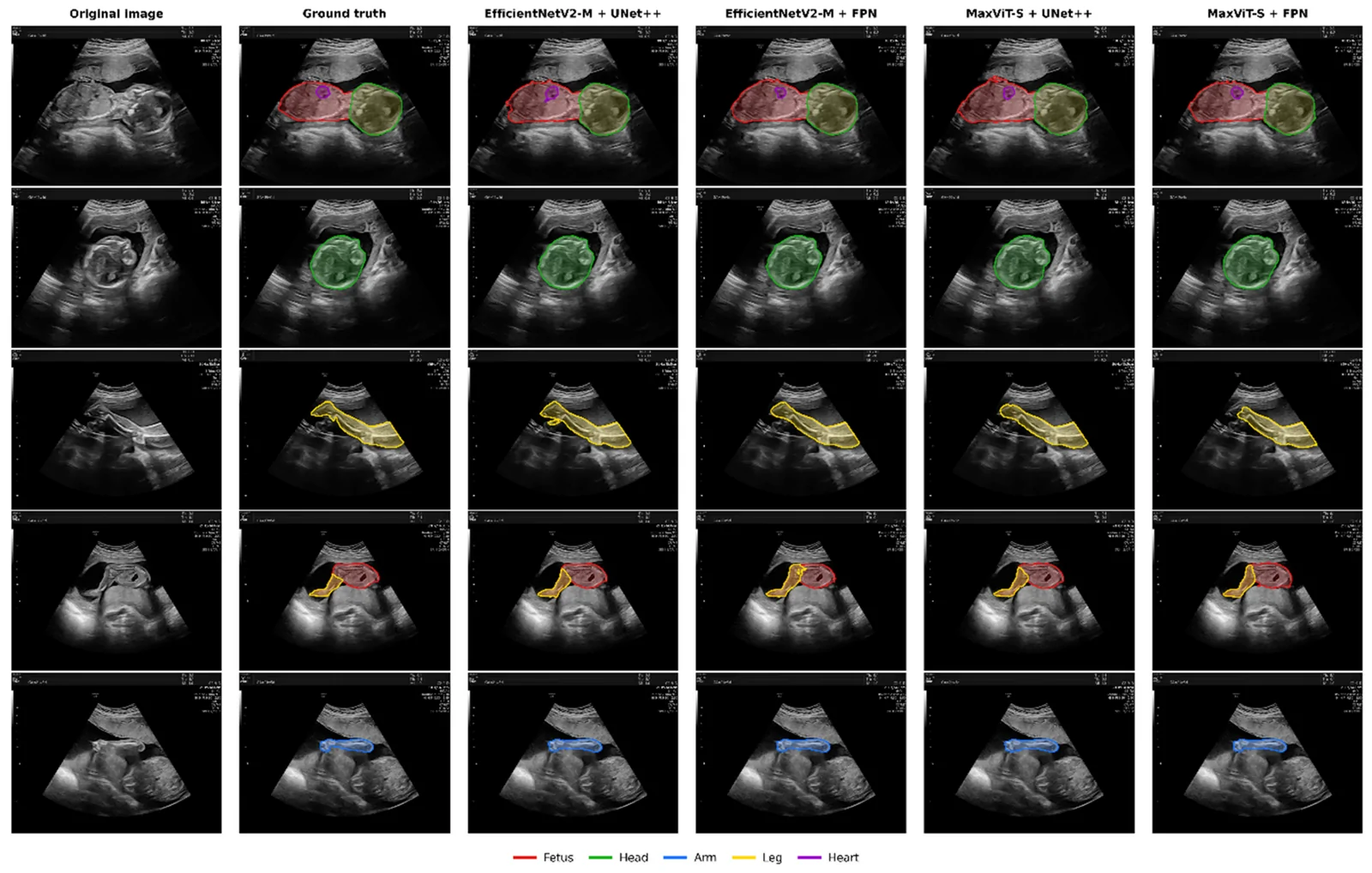
Qualitative comparison of fetal and sub-anatomical structure segmentation results on representative images from the test set. From left to right: original ultrasound image, expert ground-truth annotation, and predictions of EfficientNetV2-M + UNet++, EfficientNetV2-M + FPN, MaxViT-S + UNet++, and MaxViT-S + FPN. Fetus, head, arm, leg, and heart are shown in red, green, blue, yellow, and purple, respectively.

To complement the quantitative evaluation, representative images from the test set were selected to visually compare the expert annotations with the predictions generated by the four FetalSegNet configurations, as shown in Figure 8. The images in each row represent different ultrasound scanning planes and anatomical compositions. The columns show the original ultrasound image, the corresponding ground-truth annotation and the outputs of the four hybrid models. In the segmentation overlays, the fetus, head, arm, leg and heart are represented by red, green, blue, yellow and purple, respectively. The qualitative results are generally consistent with the quantitative findings presented in Table 5. Large, clearly visible structures—particularly the fetal body and head—were segmented with relatively smooth, spatially coherent boundaries by all four configurations. The predicted masks showed substantial spatial overlap with the expert annotations despite differences in image appearance and anatomical configuration. This is particularly evident in head-dominant examples, where the segmentation boundaries produced by the four models closely follow the corresponding ground-truth regions. More significant differences were observed in smaller, thinner or less clearly defined structures, such as the legs and heart. In these cases, minor boundary deviations, partial under-segmentation and localized over-segmentation can be seen in some of the predictions. These differences are more apparent visually when the target structure accounts for only a small portion of the image or has relatively weak contrast with the surrounding tissue. The heart, in particular, exhibits greater variation in appearance and spatial definition across ultrasound planes, making precise delineation of its boundaries more challenging. Of the four configurations, EfficientNetV2-M + UNet++ generally produced predictions that showed the closest visual correspondence with the expert annotations, particularly in terms of boundary continuity and preservation of the overall anatomical shape.

In addition to the overall performance, the DSC scores were examined individually for the fetal, head, arm, leg and heart classes, in order to analyze differences in segmentation performance across anatomical structures. The class-wise results are provided in Table 5. The highest DSC score for the head was obtained by EfficientNetV2-M + UNet++ (0.9677). This consistently high performance suggests that the relatively large size of the fetal head and its well-defined anatomical boundaries contributed to accurate segmentation across the evaluated architectures. The highest fetus class value was obtained by MaxViT-S + FPN (0.9526), followed by MaxViT-S + UNet++ (0.9437), EfficientNetV2-M + FPN (0.9317) and EfficientNetV2-M + UNet++ (0.9247). For the arm class, the highest DSC score (0.9488) was achieved by EfficientNetV2-M + UNet++, whereas the corresponding values for EfficientNetV2-M + FPN, MaxViT-S + UNet++ and MaxViT-S + FPN were 0.9068, 0.9050 and 0.8702, respectively. This variation suggests that segmentation of the arm was more sensitive to the selected encoder–decoder configuration than segmentation of larger anatomical regions. For the leg class, EfficientNetV2-M + UNet++ produced the highest value (0.8803), while MaxViT-S + UNet++ produced the lowest (0.8221). For the heart, the DSC scores were relatively close across the four configurations, ranging from 0.8837 to 0.8982. EfficientNetV2-M + FPN achieved the highest DSC score for the heart (0.8982). This suggests that all four configurations exhibited comparable segmentation capability for the heart. However, the lower DSC values compared with those for the head and fetus indicate the greater challenge associated with delineating this smaller anatomical structure. Analysis of the data by class clearly shows a relationship between anatomical size, boundary visibility and segmentation performance.

The complete absence of an anatomical structure from the predicted segmentation, as well as the generation of a structure absent from the reference annotation, were evaluated separately from the region-overlap metrics. The presence of a meaningful predicted region was considered a positive indication for each anatomical class, and the model predictions were compared with the corresponding presence status in the reference annotations. Precision, Recall, Specificity, the F1-score and Accuracy were calculated independently for each anatomical class on the test set, and the results are presented in Table 6. On average, the F1-scores of the four configurations across the five anatomical classes ranged from 0.9339 to 0.9473, while the corresponding accuracy values ranged from 0.9614 to 0.9691. These results suggest that all four configurations were effective at identifying the presence of target anatomical structures in ultrasound images. The anatomical presence analysis shows that the models achieved a high level of recall for most structures. The main reduction in presence-classification performance was associated with precision for the arm class. This finding is consistent with the qualitative segmentation results, in which localized false-positive regions were occasionally observed for the arm. Notably, the high F1-score and accuracy values across most classes suggest that the observed segmentation challenges for smaller or less distinct structures were not primarily due to an inability to detect their presence, but rather to the precision of their spatial delineation.

**Figure 9:**
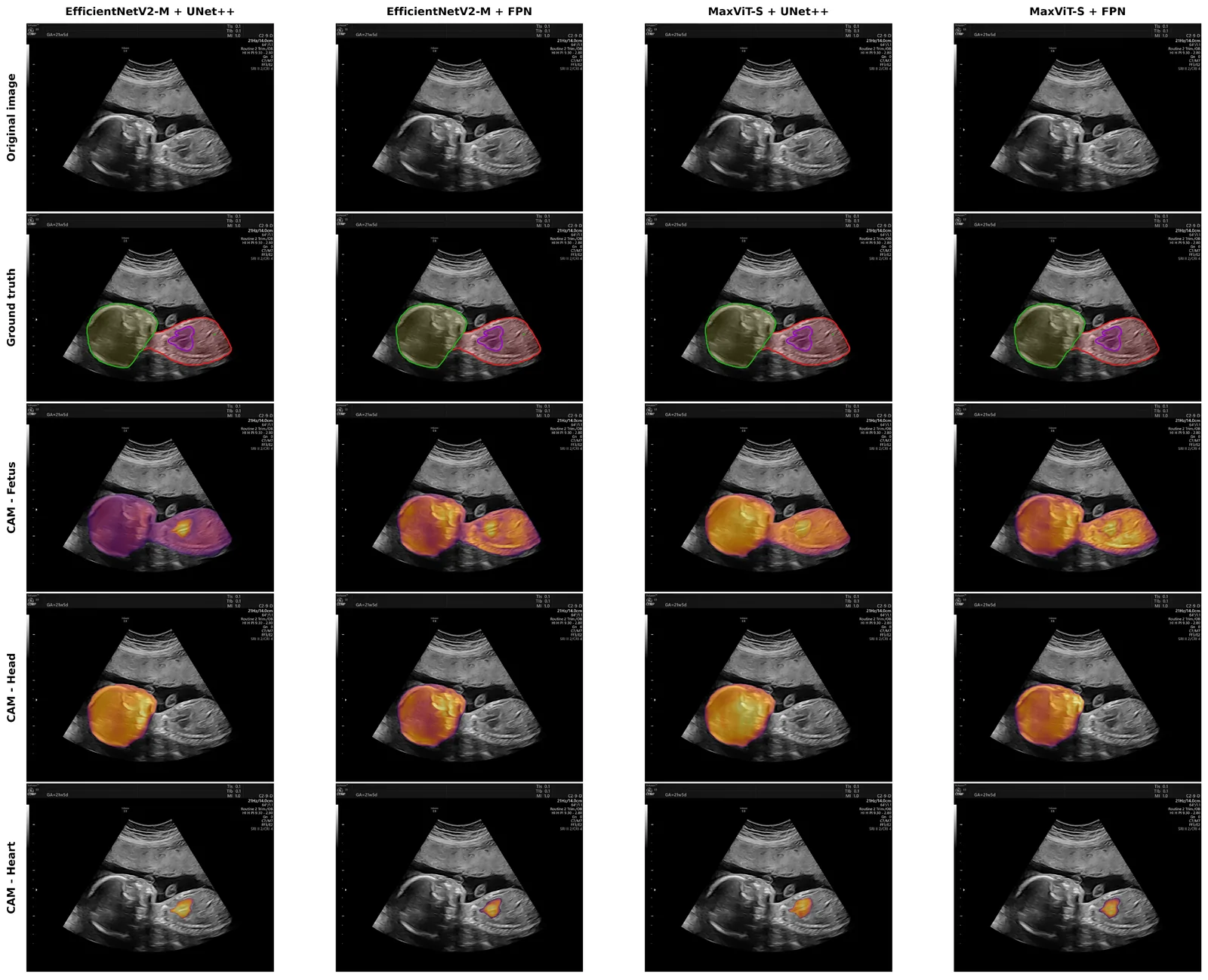
HiResCAM-based explainability analysis of the four FetalSegNet configurations. Columns correspond to EfficientNetV2-M + UNet++, EfficientNetV2-M + FPN, MaxViT-S + UNet++, and MaxViT-S + FPN. Rows show the original ultrasound image, expert ground-truth annotation, and class-specific HiResCAM activation maps. The activation maps illustrate the image regions contributing to the segmentation predictions for the corresponding anatomical structures.

To investigate which image regions contribute to the segmentation decisions of the proposed models, a high-resolution, spatially faithful variant of Grad-CAM called HiResCAM was employed [31,42]. Class-specific activation maps were generated for each anatomical structure independently. As the task was segmentation rather than classification, HiResCAM was applied in a manner adapted for segmentation. For each structure, a scalar target was defined as the sum of the positive pre-sigmoid logits of the corresponding output channel across the image. Its gradient was then taken with respect to the activations of the final decoder feature map (the input to the per-class segmentation head). This was then multiplied by the activations and their gradients element-wise, summed over the feature channels, and passed through a ReLU. The gradients were used directly rather than being spatially pooled. As all four configurations expose the same segmentation-head interface, an identical target layer was used for each encoder–decoder pair. This makes the maps directly comparable across models. The proportion of the activation-map energy falling within the corresponding expert-annotated region was quantified using an energy-ratio measure. As the four configurations had different input dimensions, the activation maps were first resized to their respective padded input dimensions. They were then cropped to the original 864 × 1136 pixel image region and spatially aligned with the expert masks. Figure 9 shows the qualitative HiResCAM results, with the columns representing the four FetalSegNet configurations and the rows displaying the original ultrasound image, expert annotations and class-specific activation maps. Overall, the activation maps were mostly concentrated within or around the relevant anatomical regions, suggesting that the models primarily relied on spatially relevant image information when generating their segmentation predictions. The activation patterns were particularly well localized to the fetal head and body regions, which also demonstrated relatively high segmentation performance in the quantitative evaluation. Differences between the four configurations were also observable in the spatial distribution and compactness of the activation maps. EfficientNetV2-M + UNet++ generally produced spatially coherent activation patterns that closely followed the corresponding anatomical regions, particularly for larger structures. The MaxViT-S-based configurations also demonstrated anatomically relevant activation patterns, though some examples showed activation that extended into neighbouring regions. HiResCAM analysis provides additional evidence that the FetalSegNet models focused primarily on anatomically relevant image regions rather than relying on unrelated background information. Correspondence between activation regions and expert annotations was strongest for relatively large, clearly delineated structures, whereas more diffuse, boundary-extending activations were observed for smaller, less clearly defined structures. These findings are consistent with the class-wise segmentation results and support the use of HiResCAM as a complementary interpretability analysis tool for the proposed multi-label fetal ultrasound segmentation framework.

The anatomical consistency of the HiResCAM explanations was assessed quantitatively using the proportion of activation energy located within the corresponding region annotated by experts. The class-wise and model-wise HiResCAM energy ratios, obtained across the complete unseen test set, are given in Table 7. Higher energy ratios indicate that a greater proportion of the model’s activation was concentrated in the corresponding anatomical region. The HiResCAM energy analysis shows that a large amount of the energy used to activate the model was concentrated within the expert-defined anatomical regions relevant to each configuration. The highest and most consistent energy ratios were observed in the fetus and head, while the leg exhibited the greatest reduction and inter-model variability. These findings are consistent with quantitative and qualitative segmentation analyses, providing complementary evidence that the models generally relied on anatomically relevant spatial information when generating segmentation predictions.

## 4. Discussion

Prenatal ultrasound segmentation is challenging because fetal anatomy can vary substantially in terms of appearance, size, orientation and visibility across scanning planes. In addition, the relatively low contrast and inconsistent texture of ultrasound images can make delineating anatomical boundaries difficult, especially for small or elongated structures. In this study, these challenges were addressed through a unified multi-label segmentation formulation in which the fetus, head, arms, legs, and heart were represented by five independent mask channels. This approach was particularly appropriate as sub-anatomical structures can overlap with the overall fetal mask, meaning they cannot be adequately represented by mutually exclusive semantic classes. This approach was suitable for independently annotated anatomical regions that may overlap spatially, since it is not possible to directly preserve multiple labels associated with the same pixel using mutually exclusive class assignments. While all five channels were optimized within a shared segmentation network, no explicit anatomical consistency constraints were imposed on their predictions. While this design allows potentially overlapping structures to be represented independently, it does not guarantee anatomically consistent relationships between the predicted masks. Using patient-level data separation ensured that images from the same patient were not distributed across the training, validation and test sets. This provided a more independent assessment of generalization.

All four of the evaluated encoder–decoder configurations produced consistently high segmentation results on the independent test set. The EfficientNetV2-M + UNet++ configuration achieved the highest DSC score (0.9232) and Intersection over Union (IoU) score (0.8746), as well as a precision score of 0.9393 and a recall score of 0.9248. Critically, this performance was also supported by boundary-based metrics, achieving the lowest HD_95_ (4.23 mm) and ASSD (1.02 mm) of all the evaluated models. The observed differences in performance between the models may be related to the complementary characteristics of the selected architectures. EfficientNetV2-M provides efficient hierarchical feature extraction, whereas UNet++ employs nested skip connections to facilitate the progressive recovery and integration of spatial information. This combination may be particularly useful for ultrasound segmentation, where accurate localization and boundary identification are important. The FPN-based configurations also achieved high overall performance, but their boundary-distance measures were generally higher than those of the EfficientNetV2-M + UNet++ configuration. While the MaxViT-S-based models produced competitive results, they did not consistently outperform the EfficientNetV2-M configurations. These findings suggest that incorporating a transformer-based encoder alone does not necessarily improve the segmentation of all fetal structures, and that the interaction between the encoder and decoder architectures is also important.

A class-based analysis revealed significant differences in anatomical structures. The head achieved the highest DSC scores consistently across all four models. This relatively stable performance is likely due to the comparatively large and recognizable morphology of the fetal head, as well as its more distinguishable boundary in the evaluated images. In contrast, the leg was the most challenging structure. This lower performance is consistent with the structure’s anatomical characteristics: fetal legs are relatively thin and elongated, may be partially visible, and can exhibit weak or ambiguous boundaries depending on the scanning plane. Another important observation is provided by the arm class. While the models generally achieved a high recall rate for the arm class, the precision rate was comparatively lower. This indicates that the models could detect most images containing an arm, but would occasionally generate an arm prediction in a region where the structure was not present. This pattern is particularly relevant for multi-label segmentation, as the independent representation of each structure enables a model to produce a class-specific prediction, even when that structure is absent from the image. Similar, although less pronounced, challenges were observed for the heart. The heart may present particular challenges due to its relatively small size and variation in appearance and position across ultrasound examinations. The segmentation of the fetal heart requires particular consideration due to its importance in prenatal assessment and the difficulties involved in identifying cardiac structures in ultrasound images. In this study, the heart was treated as an anatomical target for segmentation rather than as an indicator of cardiac health or disease. Consequently, the reported segmentation metrics quantify the correspondence between the predicted cardiac region and the expert-provided reference masks. They do not measure the detection of congenital heart disease, the characterization of cardiac structural abnormalities or the assessment of fetal hemodynamics status. Furthermore, the reference annotations were not accompanied by a formal multi-annotator agreement analysis. Therefore, variability in cardiac visibility, image quality and boundary delineation may influence the measured segmentation performance, particularly when the heart is only partially visible or its boundaries are indistinct. Further validation would be required to establish whether the outputs of the segmentation can reliably support subsequent cardiac assessment tasks. These considerations are essential before the proposed framework can be extended to applications involving fetal cardiac screening or clinical decision-making.

The HiResCAM analysis offered a supplementary view of the learned representations. The high energy ratios observed within the expert-defined anatomical regions suggest that the activation patterns were generally spatially aligned with the target structures. The fetus and head exhibited particularly high energy ratios. This finding is consistent with their high segmentation performance and suggests that the models mainly focused on relevant anatomical regions when making their predictions. In contrast, the leg showed lower and more variable energy ratios, consistent with its lower DSC performance and the difficulty in delineating its thin, extended morphology. It is important to note that the HiResCAM results should be interpreted as complementary evidence of spatial alignment rather than as definitive proof that the models use clinically appropriate reasoning.

Although the dataset was derived from ultrasound videos, FetalSegNet processes each frame independently, without taking into account temporal information from adjacent frames. In clinical ultrasound examinations, anatomical assessment often involves successive observations and adjustments according to fetal position, anatomical visibility and image quality. Consequently, the current framework cannot distinguish between structures outside the imaging plane and those that are insufficiently visualized due to image quality or unclear anatomical boundaries. Incorporating temporal feature fusion mechanisms, such as ConvLSTM, could improve segmentation stability by utilizing information from neighbouring frames. Reinforcement learning (RL) could provide an additional approach to addressing these dynamic characteristics, enabling adaptive decisions based on previous observations and task-specific feedback. Future studies could investigate integrating an RL-based decision module with FetalSegNet to support the selection of informative frames, localization of anatomical regions, or the iterative refinement of segmentation predictions. This could extend the framework to sequential ultrasound analysis while preserving its multi-label anatomical representation.

Several limitations need to be considered when interpreting these findings. Firstly, the dataset was obtained from a single source, which may restrict how widely the models can be applied to different clinical centers, ultrasound systems, acquisition protocols and patient populations. In clinical terms, this domain shift could render the segmentations and biometrics derived from them unreliable when applied to unseen scanners or populations. To address this limitation, future studies could investigate test-time augmentation, domain-randomized data augmentation and semi-supervised adaptation using unlabeled ultrasound images acquired from different devices. These strategies could improve robustness to variations in equipment and acquisition conditions. Secondly, the physical pixel spacing used for HD_95_ and ASSD was estimated from the 1 cm depth-scale markings embedded along the left side of the ultrasound images rather than being obtained directly from the metadata. As fetal biometric indices, and the gestational age and growth assessments based on them, are reported in millimeters, calibration errors could affect these clinically used measurements. While this approach allows for distance measurements in physical units, it may introduce uncertainty in the measurements compared to acquiring pixel spacing information directly from the original imaging metadata. Thirdly, some anatomical structures, particularly smaller or less consistently visible ones, were represented less frequently than larger fetal regions. This imbalance may contribute to the observed differences in class-specific performance. From a clinical perspective, this implies lower reliability for structures such as the limbs and heart, which are relevant to skeletal and cardiac screening. Therefore, predictions for these structures warrant additional caution. Additionally, while the reference masks were generated under expert supervision, the level of inter-annotator agreement and annotation uncertainty were not quantitatively assessed. For anatomical structures with poorly defined boundaries, such as the limbs and heart, DSC values calculated against a single reference mask may be influenced by annotation variability. Future studies could incorporate independent annotations from multiple experts and investigate soft or probabilistic labels to better characterize anatomical boundary uncertainty. Fourthly, the current evaluation focused on image-level segmentation and explainability, rather than assessing the effect of the generated masks on subsequent clinical measurements or decisions. Finally, the current framework does not incorporate reinforcement learning-based sequential decision-making. This limits its scope to independent, image-level analysis, with no adaptive frame selection or prediction refinement.

## 5. Conclusions

In this study, FetalSegNet, a hybrid deep learning framework, was presented for simultaneous multi-label segmentation of the fetus and sub-anatomical structures in prenatal ultrasound images. The proposed formulation represents the fetus, head, arms, legs and heart through independent mask channels, enabling anatomically overlapping structures to be segmented simultaneously without imposing mutually exclusive class boundaries. A dataset of 3321 ultrasound images from 217 patients was used to evaluate four hybrid encoder–decoder configurations based on EfficientNetV2-M or MaxViT-S combined with UNet++ or FPN. The configurations were evaluated on an independent test set of 524 images using patient-level separation. All four configurations achieved high overall segmentation performance. EfficientNetV2-M + UNet++ achieved the highest DSC score (0.9232) and IoU score (0.8746), as well as the lowest HD_95_ score (4.23 mm) and ASSD score (1.02 mm). A class-wise analysis revealed particularly strong performance for the fetal head and the fetus itself. However, the leg and, to a lesser extent, the heart presented greater challenges due to their smaller size, morphology, visibility and boundary characteristics. The anatomical presence analysis showed high F1-scores and accuracy across the evaluated structures. However, the relatively lower precision observed for the arm class indicated a tendency towards localized false-positive predictions in some images. HiResCAM analysis provided additional evidence that the models concentrated their activation energy predominantly within the corresponding anatomical regions, achieving particularly high spatial alignment for the fetus and head. The lower, more variable energy concentration observed in the leg region was consistent with its comparatively poorer segmentation performance.

Conclusively, these findings demonstrate the technical feasibility of multi-label segmentation of multiple fetal and sub-anatomical structures in prenatal ultrasound images, including cases with spatially overlapping annotations. Combining quantitative segmentation metrics, anatomical presence assessment, qualitative visualization and explainability analysis provides a multidimensional evaluation of the proposed framework. However, the single-source dataset, the indirect estimation of physical pixel spacing and the relatively limited representation of certain anatomical structures highlight the need for external validation and more extensive datasets before the framework can be considered for clinical use. Future research should therefore focus on multi-center validation, diverse ultrasound systems, improved representation of small anatomical structures, spatial calibration and evaluation of the framework in downstream fetal biometric and clinical applications.

## Figures and Tables

**Table 1:** Distribution of the prenatal ultrasound dataset according to patients, images, and annotated anatomical structures.

Subset	Number of Images	(%)	Number of Patients	Fetus	Head	Arm	Leg	Heart
Training	2297	69.17	160	548	756	620	678	799
Validation	500	15.05	28	107	151	121	136	185
Test	524	15.78	29	145	175	130	152	187
Total	3321	100.0	217	800	1082	871	966	1171

**Table 2:** Common training parameters used for all FetalSegNet configurations.

Training Parameter	Setting
Loss function	0.60 × E-Focal Loss + 0.40 × Dice Loss
Optimizer	AdamW
Initial learning rate	2 × 10^−4^
Learning-rate scheduler	Cosine annealing
Maximum epochs	150
Early stopping patience	25 epochs
Batch size	2
Gradient accumulation steps	4
Effective batch size	8
Weight decay	1 × 10^−2^
Output binarization threshold	0.45
E-Focal loss (γ-base/scale/α)	2.0/8.0/0.25

**Table 3:** Summary of the training process for the evaluated models.

Model	Parameters (M)	Best Epoch	Training Loss	Best Validation DSC
EfficientNetV2-M + UNet++	55.8	91	0.0090	0.9261
EfficientNetV2-M + FPN	54.0	53	0.0113	0.9210
MaxViT-S + UNet++	71.2	84	0.0094	0.9128
MaxViT-S + FPN	66.1	57	0.0117	0.9191

**Table 4:** Overall segmentation performance for four FetalSegNet configurations on the test set.

Model	DSC	IoU	Precision	Recall	HD_95_ [mm]	ASSD [mm]
EfficientNetV2-M + UNet++	0.9232	0.8746	0.9393	0.9248	4.23	1.02
EfficientNetV2-M + FPN	0.9052	0.8538	0.9298	0.9043	5.20	1.39
MaxViT-S + UNet++	0.9032	0.8502	0.9259	0.9122	5.59	1.39
MaxViT-S + FPN	0.8982	0.8392	0.9066	0.9101	6.48	1.53

**Table 5:** Class-wise DSC scores within anatomical structures for FetalSegNet models on the unseen test set.

Model	Fetus	Head	Arm	Leg	Heart
EfficientNetV2-M + UNet++	0.9247	0.9677	0.9488	0.8803	0.8943
EfficientNetV2-M + FPN	0.9317	0.9592	0.9068	0.8302	0.8982
MaxViT-S + UNet++	0.9437	0.9588	0.9050	0.8221	0.8862
MaxViT-S + FPN	0.9526	0.9557	0.8702	0.8290	0.8837

**Table 6:** Class-wise anatomical structure presence classification performance of the four FetalSegNet configurations on the test set.

Model	Class	Precision	Recall	Specificity	F1-Score	Accuracy
EfficientNetV2-M + UNet++	Fetus	0.8614	0.9862	0.9393	0.9196	0.9523
	Head	0.9828	0.9771	0.9914	0.9799	0.9866
	Arm	0.7922	0.9385	0.9188	0.8592	0.9237
	Leg	0.9236	0.9539	0.9677	0.9385	0.9637
	Heart	0.9684	0.9840	0.9822	0.9761	0.9828
EfficientNetV2-M + FPN	Fetus	0.9718	0.9517	0.9894	0.9617	0.9790
	Head	0.9771	0.9771	0.9885	0.9771	0.9847
	Arm	0.8289	0.9692	0.9340	0.8936	0.9427
	Leg	0.9018	0.9671	0.9570	0.9333	0.9599
	Heart	0.9632	0.9786	0.9792	0.9708	0.9790
MaxViT-S + UNet++	Fetus	0.9718	0.9517	0.9894	0.9617	0.9790
	Head	0.9719	0.9886	0.9857	0.9802	0.9866
	Arm	0.7410	0.9462	0.8909	0.8311	0.9046
	Leg	0.9221	0.9342	0.9677	0.9281	0.9580
	Heart	0.9785	0.9733	0.9881	0.9759	0.9828
MaxViT-S + FPN	Fetus	0.9789	0.9586	0.9921	0.9686	0.9828
	Head	0.9831	0.9943	0.9914	0.9886	0.9924
	Arm	0.7531	0.9385	0.8985	0.8356	0.9084
	Leg	0.8704	0.9276	0.9435	0.8981	0.9389
	Heart	0.9735	0.9840	0.9852	0.9787	0.9847

**Table 7:** Class-wise and model-wise HiResCAM energy ratios obtained on the unseen test set.

Class	EfficientNetV2-M + UNet++	EfficientNetV2-M + FPN	MaxViT-S + UNet++	MaxViT-S + FPN
Fetus	0.9832	0.9870	0.9811	0.9825
Head	0.9860	0.9861	0.9794	0.9847
Arm	0.9710	0.9542	0.9544	0.9239
Leg	0.9170	0.8730	0.9025	0.8493
Heart	0.9379	0.9560	0.9277	0.9436

## Data Availability

The data presented in this study are available upon request from the corresponding author due to ethical restrictions.

## References

[1] Salomon L., Alfirevic Z., Berghella V., Bilardo C., Chalouhi G., Costa F.D.S., Hernandez-Andrade E., Malinger G., Munoz H., Paladini D. (2022). ISUOG Practice Guidelines (updated): Performance of the routine mid-trimester fetal ultrasound scan. Ultrasound Obstet. Gynecol..

[2] Ranger B.J., Bradburn E., Chen Q., Kim M., Noble J.A., Papageorghiou A.T. (2024). Portable ultrasound devices for obstetric care in resource-constrained environments: Mapping the landscape. Gates Open Res..

[3] American Institute of Ultrasound in Medicine (2024). AIUM practice parameter for the performance of standard diagnostic obstetric ultrasound. J. Ultrasound Med..

[4] Turkan M., Dandil E., Urfali F.E., Korkmaz M. (2025). FetalMovNet: A novel deep learning model based on attention mechanism for fetal movement classification in US. IEEE Access.

[5] Fuchs F., Houllier M., Voulgaropoulos A., Levaillant J.M., Colmant C., Bouyer J., Senat M.V. (2013). Factors affecting feasibility and quality of second-trimester ultrasound scans in obese pregnant women. Ultrasound Obstet. Gynecol..

[6] Chung J.H., Pelayo R., Hatfield T.J., Speir V.J., Wu J., Caughey A.B. (2012). Limitations of the fetal anatomic survey via ultrasound in the obese obstetrical population. J. Matern.-Fetal Neonatal Med..

[7] Tsai P.-J.S., Loichinger M., Zalud I. (2015). Obesity and the challenges of ultrasound fetal abnormality diagnosis. Best Pract. Res. Clin. Obstet. Gynaecol..

[8] Wang X., Yang X., Dou H., Li S., Heng P.-A., Ni D. (2019). Joint segmentation and landmark localization of fetal femur in ultrasound volumes. Proceedings of the 2019 IEEE EMBS International Conference on Biomedical & Health Informatics (BHI).

[9] Mohan A. (2025). Deep Learning in Obstetric Sonography: A Literature Review of Techniques, Applications, and Challenges. J. Diagn. Med. Sonogr..

[10] Zhang B., Liu H., Luo H., Li K. (2021). Automatic quality assessment for 2D fetal sonographic standard plane based on multitask learning. Medicine.

[11] Chen Z., Liu Z., Du M., Wang Z. (2021). Artificial intelligence in obstetric ultrasound: An update and future applications. Front. Med..

[12] Fiorentino M.C., Villani F.P., Di Cosmo M., Frontoni E., Moccia S. (2023). A review on deep-learning algorithms for fetal ultrasound-image analysis. Med. Image Anal..

[13] Polattimur R., Yıldırım M.S., Dandıl E. (2025). Fractal-based architectures with skip connections and attention mechanism for improved segmentation of MS lesions in cervical spinal cord. Diagnostics.

[14] Ronneberger O., Fischer P., Brox T. (2015). U-net: Convolutional networks for biomedical image segmentation. Proceedings of the International Conference on Medical Image Computing and Computer-Assisted Intervention.

[15] Ashkani Chenarlogh V., Ghelich Oghli M., Shabanzadeh A., Sirjani N., Akhavan A., Shiri I., Arabi H., Sanei Taheri M., Tarzamni M.K. (2022). Fast and accurate U-net model for fetal ultrasound image segmentation. Ultrason. Imaging.

[16] Nagabotu V., Namburu A. (2024). Precise segmentation of fetal head in ultrasound images using improved U-Net model. ETRI J..

[17] Alsayat A., Elmezain M., Alanazi S., Alruily M., Mostafa A.M., Said W. (2023). Multi-layer preprocessing and U-NET with residual attention block for retinal blood vessel segmentation. Diagnostics.

[18] Zhou Z., Rahman Siddiquee M.M., Tajbakhsh N., Liang J. (2018). Unet++: A nested u-net architecture for medical image segmentation. Proceedings of the International Workshop on Deep Learning in Medical Image Analysis.

[19] Zhou Z., Siddiquee M.M.R., Tajbakhsh N., Liang J. (2019). Unet++: Redesigning skip connections to exploit multiscale features in image segmentation. IEEE Trans. Med. Imaging.

[20] Lin T.-Y., Dollár P., Girshick R., He K., Hariharan B., Belongie S. (2017). Feature pyramid networks for object detection. Proceedings of the 2017 IEEE Conference on Computer Vision and Pattern Recognition (CVPR).

[21] Dang J., Tang X., Li S. (2023). HA-FPN: Hierarchical attention feature pyramid network for object detection. Sensors.

[22] Xi J., Chen J., Wang Z., Ta D., Lu B., Deng X., Li X., Huang Q. (2021). Simultaneous segmentation of fetal hearts and lungs for medical ultrasound images via an efficient multi-scale model integrated with attention mechanism. Ultrason. Imaging.

[23] Sun Y., Yang H., Zhou J., Wang Y. (2022). ISSMF: Integrated semantic and spatial information of multi-level features for automatic segmentation in prenatal ultrasound images. Artif. Intell. Med..

[24] Tu Z., Talebi H., Zhang H., Yang F., Milanfar P., Bovik A., Li Y. (2022). Maxvit: Multi-axis vision transformer. Proceedings of the European Conference on Computer Vision.

[25] Tan M., Le Q. (2021). Efficientnetv2: Smaller models and faster training. Proceedings of the International Conference on Machine Learning.

[26] Alzubaidi M., Agus M., Alyafei K., Althelaya K.A., Shah U., Abd-Alrazaq A., Anbar M., Makhlouf M., Househ M. (2022). Toward deep observation: A systematic survey on artificial intelligence techniques to monitor fetus via ultrasound images. Iscience.

[27] Jang J., Park Y., Kim B., Lee S.M., Kwon J.-Y., Seo J.K. (2017). Automatic estimation of fetal abdominal circumference from ultrasound images. IEEE J. Biomed. Health Inform..

[28] Wang Q., Yin J., Zhang X., Ou H., Li F., Zhang Y., Wan W., Guo C., Cao Y., Luo T. (2025). Applications of artificial intelligence in early childhood health management: A systematic review from fetal to pediatric periods. Front. Pediatr..

[29] Mahmoud R., Soliman S.H., Tahir F., Elmageed N.E.A. (2026). AI-Driven innovation in fetal ultrasound imaging: Current application, challenges and future directions. Egypt. J. Radiol. Nucl. Med..

[30] Chen J., Liu P., Yang X., Wang X., Wu X., Liu Z., Liu S. (2026). SEG-FAUSP: Anatomical Structure Segmentation of the Standard Sections of Fetal Abdominal Ultrasounds. Bioengineering.

[31] Draelos R.L., Carin L. (2020). Use HiResCAM instead of Grad-CAM for faithful explanations of convolutional neural networks. arXiv.

[32] Özsoy H.G.T., Şen E.E., Urfalı F.E., Aydın G.A., Akyüz B., Dandıl E. (2026). Measurement-Oriented Evaluation of Deep Learning Models for Automated Fetal Head Biometry in Prenatal Ultrasound. J. Clin. Med..

[33] Buslaev A., Iglovikov V.I., Khvedchenya E., Parinov A., Druzhinin M., Kalinin A.A. (2020). Albumentations: Fast and flexible image augmentations. Information.

[34] Tan M., Le Q. (2019). Efficientnet: Rethinking model scaling for convolutional neural networks. Proceedings of the International Conference on Machine Learning.

[35] Ignacio M.J., Shin S., Jin H., Yoo S.J., Han D., Kim Y.-G. (2025). Revisiting U-Net: A foundational backbone for modern generative AI. Artif. Intell. Rev..

[36] Guo D., Wu Z., Feng J., Zou T. (2023). Multi-scale semantic enhancement network for object detection. Sci. Rep..

[37] Meng T., Ghiasi G., Mahjourian R., Le Q.V., Tan M. (2022). Revisiting multi-scale feature fusion for semantic segmentation. arXiv.

[38] Lin T.-Y., Goyal P., Girshick R., He K., Dollár P. (2017). Focal loss for dense object detection. Proceedings of the IEEE International Conference on Computer Vision.

[39] Milletari F., Navab N., Ahmadi S.-A. V-net: Fully convolutional neural networks for volumetric medical image segmentation. Proceedings of the 2016 Fourth International Conference On 3D vision (3DV).

[40] Loshchilov I., Hutter F. (2017). Fixing weight decay regularization in adam. arXiv.

[41] Taha A.A., Hanbury A. (2015). Metrics for evaluating 3D medical image segmentation: Analysis, selection, and tool. BMC Med. Imaging.

[42] Selvaraju R.R., Cogswell M., Das A., Vedantam R., Parikh D., Batra D. (2017). Grad-cam: Visual explanations from deep networks via gradient-based localization. Proceedings of the IEEE International Conference on Computer Vision.

